# Integrated multi-omics analysis reveals the positive leverage of citrus flavonoids on hindgut microbiota and host homeostasis by modulating sphingolipid metabolism in mid-lactation dairy cows consuming a high-starch diet

**DOI:** 10.1186/s40168-023-01661-4

**Published:** 2023-10-25

**Authors:** Yuchao Zhao, Shiqiang Yu, Huiying Zhao, Liuxue Li, Yuqin Li, Ming Liu, Linshu Jiang

**Affiliations:** Beijing Key Laboratory of Dairy Cow Nutrition, College of Animal Science and Technology, Beijing University of Agriculture, Beijing, 102206 China

**Keywords:** Dairy cows, Metaproteomics, Hindgut fermentation, Systemic inflammation, Sphingolipid metabolism

## Abstract

**Background:**

Modern dairy diets have shifted from being forage-based to grain and energy dense. However, feeding high-starch diets can lead to a metabolic disturbance that is linked to dysregulation of the gastrointestinal microbiome and systemic inflammatory response. Plant flavonoids have recently attracted extensive interest due to their anti-inflammatory effects in humans and ruminants. Here, multi-omics analysis was conducted to characterize the biological function and mechanisms of citrus flavonoids in modulating the hindgut microbiome of dairy cows fed a high-starch diet.

**Results:**

Citrus flavonoid extract (CFE) significantly lowered serum concentrations of lipopolysaccharide (LPS) proinflammatory cytokines (TNF-α and IL-6), acute phase proteins (LPS-binding protein and haptoglobin) in dairy cows fed a high-starch diet. Dietary CFE supplementation increased fecal butyrate production and decreased fecal LPS. In addition, dietary CFE influenced the overall hindgut microbiota’s structure and composition. Notably, potentially beneficial bacteria, including *Bacteroides*, *Bifidobacterium*, *Alistipes*, and *Akkermansia*, were enriched in CFE and were found to be positively correlated with fecal metabolites and host metabolites. Fecal and serum untargeted metabolomics indicated that CFE supplementation mainly emphasized the metabolic feature “sphingolipid metabolism.” Metabolites associated with the sphingolipid metabolism pathway were positively associated with increased microorganisms in dairy cows fed CFE, particularly Bacteroides. Serum lipidomics analysis showed that the total contents of ceramide and sphingomyelin were decreased by CFE addition. Some differentially abundant sphingolipid species were markedly associated with serum IL-6, TNF-α, LPS, and fecal *Bacteroide*s. Metaproteomics revealed that dietary supplementation with CFE strongly impacted the overall fecal bacterial protein profile and function. In CFE cows, enzymes involved in carbon metabolism, sphingolipid metabolism, and valine, leucine, and isoleucine biosynthesis were upregulated.

**Conclusions:**

Our research indicates the importance of bacterial sphingolipids in maintaining hindgut symbiosis and homeostasis. Dietary supplementation with CFE can decrease systemic inflammation by maintaining hindgut microbiota homeostasis and regulating sphingolipid metabolism in dairy cows fed a high-starch diet.

Video Abstract

**Supplementary Information:**

The online version contains supplementary material available at 10.1186/s40168-023-01661-4.

## Introduction

To satisfy their requirements for glycogenic precursors such as propionate, the diets of high-yielding dairy cows often contain considerable amounts of starch-containing concentrates. However, excessive grain or starch feeding might result in varying degrees of rumen acidosis, a common metabolic disorder that impairs cows’ production and health [1]. While ruminal health has garnered great attention, there is emerging evidence that the hindgut and its resident bacteria are crucial for ruminant health and production efficiency [2–4]. However, when ruminants are fed high amounts of grain diets, approximately 50% of the dietary starch escapes from the rumen to the lower gastrointestinal tract where it is digested [5]. Depressed hindgut pH can damage mucosal permeability and integrity [6], contributing to systemic inflammation to some extent in dairy cows [7, 8]. Gut dysbiosis changes the composition and reduces microbiota functionality by promoting the proliferation of opportunistic bacteria (e.g., faster growers) and disintegration and lysis of those that cannot adapt to suboptimal hindgut environments [9].

Nutritional strategies, such as using phytogenic feed additives have been shown to benefit the overall health and performance of ruminant livestock by regulating the balance of gastrointestinal tract microbiota [10]. It is well-known that plant flavonoids are anti-inflammatory and anti-oxidative polyphenols. The *citrus* genus is one of the most important fruit crops for food processing and fresh juice production worldwide [11]. The most important metabolites of the *Citrus* genus are flavonoids, mainly hesperidin, naringin, nobiletin, and tangeretin [12]. Previous studies demonstrated that dietary supplementation with citrus flavonoid extract reduces rumen inflammation and improves ruminal function in Holstein bulls [13, 14], beef heifers [15], or fattening goats [16] fed high-concentrate diets. In a recent study, we found that feeding citrus flavonoids to mid-lactation dairy cows altered the rumen environment, including the microbial population and metabolites, and had a beneficial effect on milk yield [17].

Generally, phytochemicals are characterized by low bioavailability. In humans and rodents, only 5–10% of dietary polyphenols are directly absorbed in the small intestine, while the rest travel intact to the large intestine, where they are metabolized by the gut microbiome [18]. In monogastric animals, citrus flavonoids also have limited bioavailability after oral administration, leaving the majority unabsorbed and persisting in the colon [19, 20]. Recent studies have highlighted the important role of the gut microbiota and in vivo biotransformation on the bioactivity of citrus flavonoids [19, 21]. For ruminants, it is unknown whether citrus flavonoids are absorbed across the rumen epithelium. It was reported that rumen bacteria are known to have the ability of partially deglycosylating naringin and hesperidin [22]. Dietary flavonoids are well-known to have high affinity to bind dietary proteins and other biomolecules [23]. Therefore, flavonoids not involved in biotransformation in the rumen can flow into the lower gastrointestinal tract with undigested nutrients. The unmodified citrus flavonoid compounds accumulate in the large intestine, where they are broken down by the gut microbes to supply other biologically active substances.

Hindgut microbiota can degrade about 17% of the digested cellulose and produce approximately 12% of the total short-chain fatty acid (SCFA) for ruminant livestock [24]. In the case of ruminants fed high-concentrate diets, targeting the hindgut offers a window of opportunity for further improving their health status [25]. Additionally, previous studies have suggested that the cross-talk between the hindgut microbiota and host oxidative and inflammatory responses is pivotal to provide unique insights into improving the welfare and health of cows [26, 27]. Nevertheless, few studies have examined the effect of dietary flavonoids on the hindgut microbial community and function in dairy cows.

Therefore, the objective of this study was to evaluate the effect of citrus flavonoids on the composition and functional features of the hindgut microbiota and inflammatory host-response biomarkers of lactating dairy cows fed a high-starch diet. Considering the potential interaction of gut microbiota and plant flavonoids, we hypothesized that attenuation of hindgut dysbiosis by citrus flavonoids is mediated through modulation of the hindgut microbiota, which subsequently leads to alleviation of systemic inflammation in the host.

Due to its ability to represent the hindgut and its non-invasive nature, fecal analysis is the ideal method of examining hindgut dysbiosis in dairy cows [28]. As a result of the fast-emerging sequencing technique, the microbiota of feces has gained increasing attention in the past 20 years. Because proteins are more actual and stable, metaproteomics can be expected to provide accurate and more real profiling of hindgut microbiota function [29]. Integrating omics techniques yields a better understanding and clearer picture than single omics analysis. In the present study, we investigated the anti-inflammatory activity of citrus flavonoid extract (CFE) in dairy cows fed a high-starch diet. Subsequently, we determined the changes in fecal microbiota, fecal metabolites, and serum metabolites by employing 16S rRNA gene sequencing and metabolomics. In addition, to verify the effects of flavonoids on the function of fecal microbiota, we further investigated the metaproteomics profiling.

## Methods

### Experimental design and treatments

The CFE product was obtained from Shaanxi Xiazhou Biotechnology Co., Ltd. (Xi’an, China). Citrus flavonoids were isolated from the peel powder of *Citrus reticulata Blanco*. Briefly, 1 kg of powdered orange peel was extracted twice with 15 L of a 0.1% calcium carbonate solution at 100 °C for 1.5 h. The extraction was then dried by rotary evaporation at 37 °C and under decreased pressure. The total flavonoids were enriched by AB-8 macroporous absorption resin columns and eluted with 80% ethanol (twofold column volume). The eluents were collected and concentrated to dryness for use. The total flavonoid content of CFE (56.83%) was analyzed with aluminum nitrate spectrophotometry using rutin equivalents at 510 nm. An HPLC system (1290 Infinity; Agilent Technologies, Inc.) was used to analyze the concentrations of major flavonoids in CFE, including naringin, hesperidin, neohesperidin, nobiletin, and tangeretin, based on the procedure of Jiang et al. [30]. The chemical composition of CFE was shown in Table S1.

Experimental procedures were approved by the Institutional Animal Care and Use Committee of Beijing University of Agriculture. Eight multiparous, mid-lactation Chinese Holstein cows [(mean ± SD: 3.22 ± 1.12 parity; 154 ± 12 days in milk (DIM), 33.8 ± 3.7 kg/day of milk, 668 ± 24 kg of bodyweight)] were used as experimental animals. Cows were stratified by parity, milk production, and DIM and used in a replicated (4 squares) 2 × 2 Latin square design with 28-day feeding periods. Cows were assigned randomly to one of the two treatments, either control (CON) or supplemented with 100 g/day CFE. The dose of CFE was based on our previous study using mid-lactation dairy cows [17]. Each experimental period consisted of 21 days for adaptation to the diet and 7 days for sampling.

Cows were housed in individual tie stalls and had free access to water. Diets were formulated to meet the requirements of cows according to the NRC [31]. The ingredient and nutritional level of the total mixed ration (TMR) containing a 40:60 forage to concentrate ratio was shown in Table S2. The starch content of TMR was determined using a commerical kit (Beijing Boxbio Science ＆ Technology Co.,Ltd., Beijing, China). Cows were moved into the tie stall barn and fed the basal diet for 6 days before the start of the experimental period. At day 0, one-half of the cows were switched to the CFE treatment and the other 13 cows remained on the basal diet (CON). Cows remained on those diets for 28 days (period 1) and then were abruptly changed to the opposite treatment and fed for additional 28 days (period 2). Before the morning feeding, individual feed bunks were emptied, and the amount of the orts was recorded. The dry matter (DM) of individual feed ingredients was analyzed weekly and the basal diet was adjusted accordingly. Cows were fed at 1000 h daily at 110% expected intake, with water available ad libitum in each stall. The CFE was top-dressed with the morning feed. For cows receiving CFE top-dressed, 300 g/day of dried molasses was mixed with the dry CFE product to ensure consumption by cows, and all cows readily consumed it. A similar amount of dried molasses was top-dressed to all cows. All cows were milked three times daily at 0700, 1330, and 2000 h, and milk production was recorded daily throughout the duration of the experiment.

### Serum biochemical parameter analysis

Blood samples were collected from the coccygeal region using venipuncture, at 0800 h, on the last day of each sampling period. Blood samples were collected into evacuated tubes containing coagulants (Vacutainer, Becton Dickinson, Franklin Lakes, NJ). Samples were kept at room temperature until serum was separated by centrifugation at 3500 × *g* at 15 °C for 15 min. Serum aliquots were stored at − 80 °C for quantification of biochemical parameters and metabolome analysis. Serum levels of aspartate aminotransferase (AST, #BC1565) and alanine aminotransferase (ALT, #BC1555) were determined using commercially available kits (Beijing Solarbio Science & Technology Co., Ltd., Beijing, China). In addition, serum levels of total protein (TP, #A045-4–2), albumin (ALB, #A028-2–1), IL-1β (#H002-1–2), IL-6 (#H007-1–1), IL-10 (#H009-1–2), TNF-α (#H052-1–2), lipopolysaccharide (LPS, #H255-1–1), LPS-binding protein (LBP, #H253), serum amyloid A protein (SAA, #H134), and haptoglobin (Hp, #H136) were analyzed with ELISA kits (Nanjing Jiancheng Bioengineering Institute, Nanjing, China). All the above biochemical parameters were determined according to the manufacturer’s instructions. Absorbance was measured with a microplate reader (Multiskan FC; Thermo Fisher, New York, NY).

### Fecal SCFA and LPS determination

Fecal grab samples were taken from cows by stimulating defecation or directly from the rectum. Fecal samples were composited by animal according to fresh weight over the last 3 days of each period and then frozen at − 80 °C. Fecal SCFA were analyzed using the method of Petri et al. [32] with minor modifications. Briefly, 2 g of thawed feces from each sample was mixed with 2 mL of distilled water. Then, 600 μL of the internal standard 4-methylvaleric acid (Sigma-Aldrich, Saint Louis, MO) and 0.4 mL of 25% metaphosphoric acid were added to the suspension. Those mixtures were centrifuged at 15,000 × *g* at 4 °C for 20 min. Fecal SCFA were separated and quantified using a gas chromatograph (7890B; Agilent Technologies, Inc.) with a capillary column (30 m × 0.250 mm × 0.25 μm; DB-FFAP; Agilent Technologies, Inc.) and flame-ionization detection. The injector and detector temperatures were 225 and 250 °C, respectively. The concentrations of LPS in feces were determined using an ELISA commercial kit (#hj-C15344, Lanpaibio, Shanghai, China) according to the manufacturer’s instructions.

### Serum and fecal metabolome

Serum and fecal metabolites were extracted according to the procedures of Guo et al. [33] and Luo et al. [34]. Metabolic profiles were generated using an untargeted method with an ultrahigh-performance liquid chromatography‐tandem mass spectrometry platform (UPLC‐MS/MS). The LC separation was performed with a UPLC BEH Amide column (2.1 mm × 100 mm, 1.7 μm, Waters). The mobile phase was composed of buffers: (A) water with 0.1% formic acid and (B) acetonitrile with 0.1% formic acid. The flow rate was 0.3 mL/min, and the injection volume was 1 μL. An increasing linear gradient was 5% B for 0 to 2 min, 5 to 95% B for 2 to 12 min, 95% B for 12 to 15 min, and 95 to 5% B for 15 to 17 min—data collected in both positive and negative ion mode.

The LC‒MS data were processed with Progenesis QI software (Waters, Wilmslow, UK). We generated a multivariate data matrix containing sample identity, ion identity (retention time and m/z), and ion abundance after centroiding, deisotoping, filtering, peak recognition, and integration. Metabolites were annotated using accurate mass matching with the Human Metabolome Database (HMDB) and Kyoto Encyclopedia of Genes and Genomes (KEGG). The processed data matrix was exported into SIMCA-P software (version 13.0, Umetrics AB, Umea, Sweden), transformed by Pareto scaling, and then characterized by multivariate data analysis. Pathway analysis was performed using MetaboAnalyst (McGill University, Quebec, CA; http://metaboanalyst.ca).

### Serum lipidome analysis

Serum lipid extraction was performed according to the description of Xia et al. [35]. Untargeted lipidomics analysis was performed using a UPLC coupled with a Q Exactive hybrid quadrupole Orbitrap mass spectrometer (Thermo Fisher Scientific, San Jose, CA). Reversed-phase chromatographic separation was employed with a UPLC BEH C8 column (2.1 mm × 100 mm, 1.7 μm) at 40 °C maintained at 55 °C. The mobile phase consisted of (A) acetonitrile/H_2_O (60: 40, v/v) containing 10 mM ammonium formate and (B) acetonitrile/isopropanol (10: 90, v/v) containing 10 mM ammonium formate. The gradient was set as follows: 0 to 23 min, 30 to 98% B; 23 to 30 min, 98% B; 30 to 35 min, 98 to 30% B; followed by 4 min of re-equilibration of the column before the next run. The flow rate was 0.26 mL/min, and the injection volume was 1 μL. Mass spectrometry analysis was performed in both positive and negative ion modes. Lipid species were identified and quantified via Lipidsearch software (version 4.1.16; Thermo Fisher Scientific, Waltham, MA).

### Fecal DNA extraction, 16S rRNA gene sequencing, bioinformatics analysis

Fecal bacterial DNA was isolated with the QIAmp Fast DNA Stool Mini Kit (#51,604; Qiagen, Hilden, Germany) based on the manufacturer’s guidelines. The quantification and quality check of the extracted DNA were performed with a Nano-Drop 2000 spectrophotometer (Thermo Fisher Scientific, DE). The 16S rRNA sequencing procedure was performed as previously reported [36]. Briefly, fecal DNA samples (10 ng) were used as templates for PCR amplification of the V3–V4 variable region with the primer pair 341F (5′-CCTAYGGGRBGCASCAG-3′) and 806R (5′-GGACTACNNGGGTATCTAAT-3). The thermocycling conditions for PCR involved a 3 min initial denaturation step at 95 °C, followed by 20 cycles including 10 s of denaturation at 98 °C, 10 s of annealing at 59 °C, 45 s of extension at 72 °C, and 2 min final extension step at 72 °C. PCR products were visualized on 2% agarose gels and purified with the QIAquick gel extraction kit (#28,704; Qiagen, Dusseldorf, Germany). The libraries were sequenced on an Illumina MiSeq platform generating 2 × 300 bp paired end reads.

Paired end reads were merged using FLASH (version 1.2.11). The raw reads were analyzed using QIIME (version 1.9.1) and bases with quality scores higher than 20 were retained for further analysis. Sequences were clustered into operational taxonomic units (OTU) at 97% similarity. All OTU were subjected to taxonomy assignment against the SILVA 16S rRNA database (version 138) using RDP classifier (version 2.13).

The raw sequence data reported in this paper have been deposited in the Genome Sequence Archive in National Genomics Data Center, China National Center for Bioinformation / Beijing Institute of Genomics, Chinese Academy of Sciences (GSA: CRA009531) that are publicly accessible at https://ngdc.cncb.ac.cn/gsa.

### Fecal metaproteomics analysis

The extraction of proteins from feces was conducted as described previously [37]. Briefly, proteins from approximately 0.6 g fecal samples were extracted by combining phenol extraction and buffer BPP (100 mmol/L EDTA,50 mmol/L Borax,1% PVPP, 1% tris-X-100,2% β-mercaptoethanol, 100 mmol/L Tris–HCl, pH 8.0), in a ball mill (FastPrep-96, MP Biomedicals, Eschwege, Germany). Precipitation of the extracted proteins was achieved by mixing 5 vol of 10% (w/v) ice-cold TCA acetone-dithiothreitol and incubating the mixture at − 20 °C for a whole night. Protein pellets were washed in ice-cold acetone-dithiothreitol, vacuum-dried, and resuspended in solubilization buffer (8 mmol/L urea, 20 mM dithiothreitol, and 1% SDS). The protein concentration was analyzed using the Bradford assay. Proteins were fractionated via SDS‒PAGE as described previously [38]. Proteins were then resuspended and digested using trypsin at a final ratio of 1:50 (trypsin-protein) overnight at 37 °C. Trypsin-digested peptides were desalted using a solid phase extraction column (Oasis HLB SPE, Waters) and dried using vacuum centrifugation. Before LC–MS/MS measurements, they were reconstituted in 20 μL of 0.1% formic acid/2% acetonitrile.

Protein digests were analyzed by LC‒MS/MS on an EASY-nLC 1200 (Thermo Fisher Scientific) coupled online for analysis with a Q Exactive Orbitrap HF-X mass spectrometer (Thermo Fisher Scientific). Trapped peptides were then separated using a C18 analytical column (75 μm × 25 cm × 2 μm; Thermo Fisher Scientific) at 40 °C. Chromatography was performed using 2% acetonitrile (plus 0.1% formic acid) (A) and 80% acetonitrile (0.1% formic acid) (B) at flow rates of 0.3 μL/min. Peptides were eluted by using a step-wise gradient: 0–1 min, 0–5% B; 1–63 min, 5–23% B; 63–88 min, 23–48% B; 88–89 min, 48–100% B; 89–95 min, 100% B.

Continuous scanning for eluted peptide ions was performed within the mass range of 300–1500 m/z. The mass spectrometer was set up in a data-dependent MS/MS mode with HCD as a fragmentation method, as described by Tanca et al. [39]. Raw spectra data files were processed with Proteome Discoverer (version 2.1; Thermo Fisher Scientific). The genomic database for spectrum searches was generated by downloading and merging protein sequences from the sequence repository UniProt of the top 20 bacterial genera found in the 16S rRNA gene sequencing data with protein sequences from the taxa Bos taurus. The Sequest HT search engine was configured with the following search parameters: trypsin (full) as the used enzyme, iodoacetamide as the Cys alkylation, oxidation (M), acetyl (protein N-terminus), Met-loss (protein N-terminus), Met-loss + acetyl (protein N-Terminus) as dynamic modifications, and carbamidomethyl (C) as static modifications. A maximum of two missed cleavage sites and a precursor mass tolerance threshold of 10 ppm were additional search parameters. The data were filtered using a 1% peptide FDR.

### Statistical analysis

Fecal SCFA and serum biochemical parameters were analyzed using the mixed model of SAS (version 9.4; SAS Institute, Cary, NC). Period and treatment were considered as fixed effects. Statistical analysis of omics data was conducted using R (version 4.0.3). All data are presented as least square means and standard error of means unless otherwise specified.

The alpha diversity indices (ACE and Shannon) between treatments were compared using the nonparametric Kruskal‒Wallis testing method with Mothur (version 1.30.2). Beta diversity was determined to compare the bacterial structure between groups with Bray‒Curtis dissimilarity and weighted UniFrac distance and visualized by PCoA. Analysis of similarities (ANOSIM) using the vegan package of R with 999 permutations was used to detect the dissimilarities between CON and CFE. Linear discriminant analysis (LDA) effect size (LEfSe) was performed to identify differentially abundant taxa. The FDR method was applied to adjust *P* values, and differences were considered as significant at *P* < 0.05.

Differentially abundant metabolites and lipid species were recognized with a multi-criteria evaluation according to the Wilcoxon rank-sum test (*P* < 0.05) and variable importance in projection (VIP) > 1 in an orthogonal partial least square discriminant analysis (OPLS-DA) model. Correlations between variables were tested by Spearman’s correlation test and meanwhile visualized by using the corrplot and pheatmap R packages. The differentially expressed proteins (DEP) were selected based on the criteria *P* < 0.05 (FDR) and fold change (FC) > 2.0 or < 0.50. All DEP were subjected to Gene Ontology (GO) analysis using UniPort’s GO annotation (https://www.uniprot.org/) to predict biological functions. Functional classifications of DEP were obtained using KEGG analysis.

## Results

### Serum biochemical parameters, fecal SCFA, and fecal LPS

**Fig. 1:**
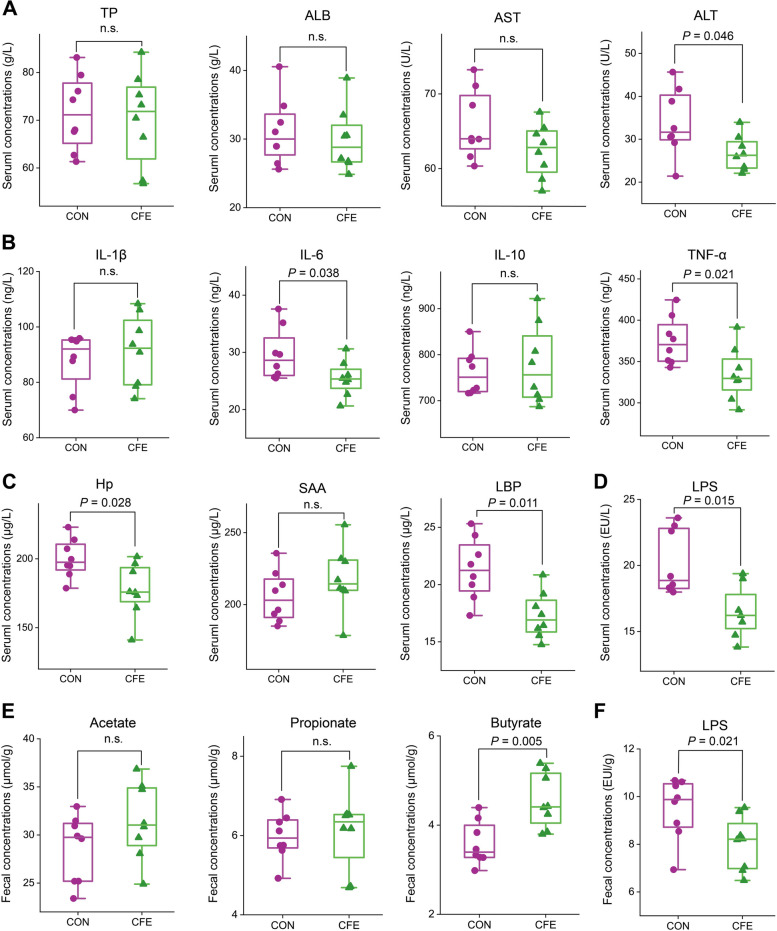
Effects of CFE on serum and fecal biochemical parameters of dairy cows. **A** Liver function biomarkers. **B** Inflammatory cytokines. **C** Inflammatory acute phase protein. **D** Serum LPS. **E** Fecal SCFA. **F** Fecal LPS. Data were presented as means ± SEM (*n* = 8 per group). CON, control; CFE, citrus flavonoid extract; SCFA, short-chain fatty acid; LPS, lipopolysaccharide; TP, total protein; ALB, albumin; AST, aspartate aminotransferase; ALT, alanine aminotransferase; Hp, haptoglobin; SAA, serum amyloid A protein; LBP, LPS-binding protein. The data are presented as the mean ± SEM

As shown in Fig. 1A–C, compared with CON, supplementing CFE decreased serum concentrations of ALT (*P* = 0.046), IL-6 (*P* = 0.038), TNF-α (*P* = 0.021), Hp (*P* = 0.028), and LBP (*P* = 0.011). We detected the major SCFA, acetate, propionate, and butyrate in the dairy cow feces. Dietary supplementation with CFE increased fecal butyrate concentration (Fig. 1D, *P* = 0.005). Supplementing CFE reduced serum LPS (*P* = 0.015) and fecal LPS (*P* = 0.021) concentrations (Fig. 1D, F). Serum TP (*P* = 0.650), ALB (*P* = 0.401), AST (*P* = 0.149), IL-1β (*P* = 0.202), IL-10 (*P* = 0.344), SAA (*P* = 0.198), fecal acetate (*P* = 0.129), and fecal propionate (*P* = 0.673) were similar between the two groups.

### Taxonomic configurations of fecal bacteria

**Fig. 2:**
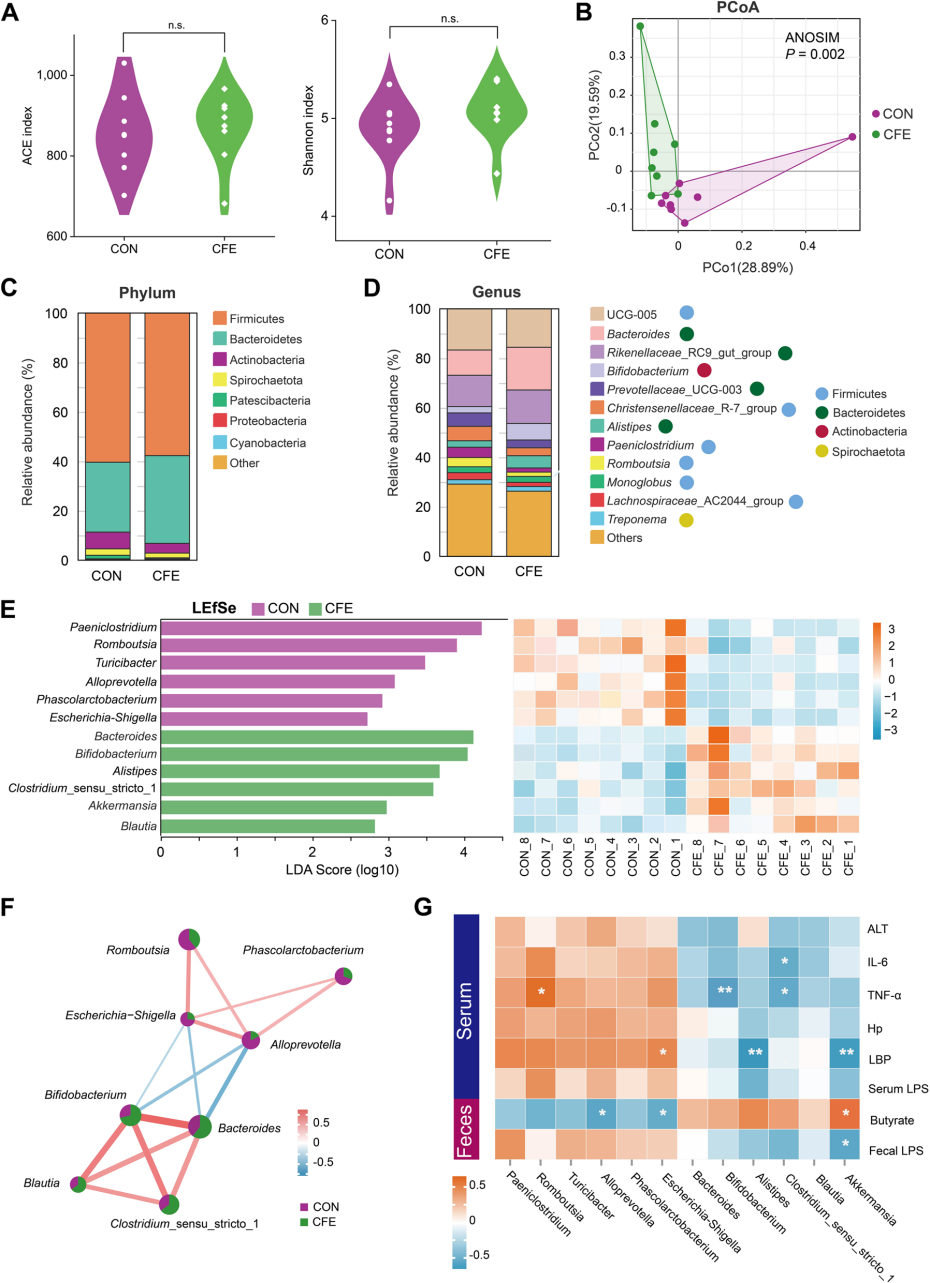
Diversity and composition of the feces bacterial community of dairy cows. **A** Alpha diversity indices. **B** Beta diversity based on the principal coordinate analysis (PCoA) using the weighted UniFrac distance. **C** Relative abundance of phyla between two groups. **D** Relative abundance of genera between two groups. **E** Analysis of differences in the microbial taxa at the genus level shown by linear discriminant analysis (LDA) effect size (LEfSe) and heatmap of differentially abundant genera. The blue color represents less abundant, red represents the more abundant (CFE vs CON). **F** Correlation network of differential genera. Red, positive correlations; blue, negative correlations. Only the genera connections (correlation values >|0.6|, *P* < 0.05) are retained. **G** Heatmap of Spearman’s correlation between fecal genera and fecal or serum biochemical parameters. Red, positive correlations; blue, negative correlations. *P < 0.05, **P < 0.01. CON, control; CFE, citrus flavonoid extract; ANOSIM, analysis of similarities; ALT, alanine aminotransferase; Hp, haptoglobin; LPS, lipopolysaccharide; LBP, LPS-binding protein

The effect of CFE on the hindgut microbiota of cows was determined by 16S rRNA high-throughput sequencing. Based on alpha diversity analysis including ACE (*P* = 0.642) and Shannon (*P* = 0.577) indices (Fig. 2A), there were no significant differences between CON and CFE. Regarding beta diversity, principal coordinate analysis (PCoA) was performed, explaining 28.9 and 19.6% of the variance, respectively, and the PCoA plot showed the clustering between CON and CFE. A significant difference was found between the two groups (weighted UniFrac, ANOSIM: *P* = 0.002; Fig. 2B). This indicates that dietary CFE significantly altered the hindgut microbial structure. As illustrated in Fig. 2C, Firmicutes, Bacteroidetes, Actinobacteria, and Spirochaetota were the dominant bacterial phyla in the two groups and accounted for more than 95% of the bacteria in the microbial communities. At the genus level, the fecal microbiome was dominated by UCG-005, Bacteroides, *Rikenellaceae* RC9_gut_group, *Bifidobacterium*, *Prevotellaceae* UCG-003, *Christensenellaceae* R-7_group, and *Alistipes* (Fig. 2D). At the phylum level, no significant differences in bacterial taxa were identified (Table S3). We also analyzed the bacterial community at the genus level using LEfSe analysis (Fig. 2E). LEfSe identified significant differences in the relative abundances of 12 genera (LDA ≥ 2.5, *P* < 0.05). Compared with CON, the relative abundances of *Bacteroides*, *Bifidobacterium*, *Alistipes*, *Clostridium*_sensu_stricto_1, *Akkermansia*, and *Blautia* in the CFE were increased. The relative abundances of *Paeniclostridium*, *Romboutsia*, *Turicibacter*, *Alloprevotella*, *Phascolarctobacterium*, and *Escherichia-Shigella* were lower in the CFE than in the CON.

We conducted Spearman’s correlation analysis (*P* < 0.05 and correlation coefficient >|0.6|) for all differential taxa at the genus level. The network diagram shows that four upregulated, and four downregulated genera are positively correlated (Fig. 2F). We further assessed the association between the differential genera and fecal or serum phenotypes by Spearman’s correlation analysis. *Akkermansia* had a strong positive correlation with fecal butyrate concentration but a significantly negative correlation with serum LBP and fecal LPS (Fig. 2G). *Clostridium*_sensu_stricto_1 and *Bifidobacterium* exhibited significant negative correlations with serum TNF-α. *Alloprevotella* and *Escherichia-Shigella* exhibited significant negative correlations with fecal butyrate. These results indicated that CFE altered the hindgut bacterial composition and metabolism, potentially decreasing systemic inflammation levels in dairy cows fed a high-starch diet.

### Alteration of fecal and serum metabolites

**Fig. 3:**
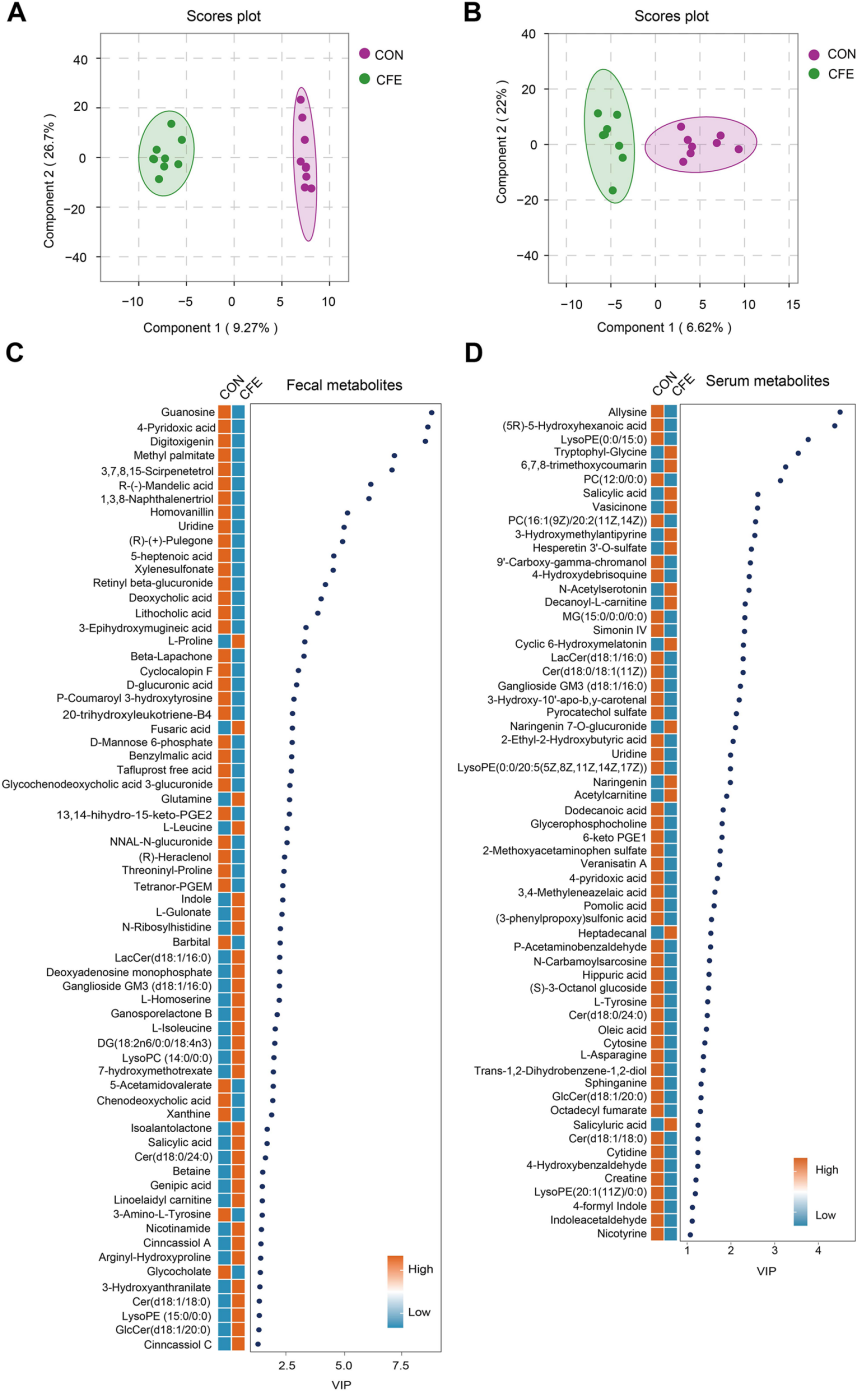
Dietary CFE caused differences in fecal metabolites and serum metabolites of dairy cows. Scores plot of orthogonal partial least square discriminant analysis (OPLS-DA) for fecal metabolites (**A**) and serum metabolites (**B**). Heatmap of differentially abundant metabolites in feces (**C**) and serum (**D**) (VIP > 1, *P* < 0.05 and |FC|≥ 1.2). CON, control; CFE, citrus flavonoid extract; VIP, variable importance in projection; FC, fold change

Next, we conducted metabolite profiling in feces and serum samples from CON and CFE based on untargeted metabolomics. We identified 685 and 528 annotated metabolites for feces and serum samples, respectively. Both score plots of OPLS-DA constructed on the fecal and serum metabolites separated the CFE group from the CON group (Fig. 3A, B), which indicated a significant change in the hindgut and host metabolites when dairy cows were fed CFE. A total of 66 fecal metabolites differed between CON and CFE, including 30 upregulated metabolites and 36 downregulated metabolites [(FC) ≥ 1.2 or ≤ 0.83, VIP > 1, and *P* ≤ 0.05] (Fig. 3C). A total of 61 serum metabolites were significantly different in CFE cows compared with CON, including 14 that were significantly greater and 47 that were lower in CFE cows (Fig. 3D). In addition, naringenin, hesperetin 3'-O-sulfate, and naringenin 7-O-glucuronide, the metabolites of citrus flavonoids, were enriched in the serum of CFE cows.

**Fig. 4:**
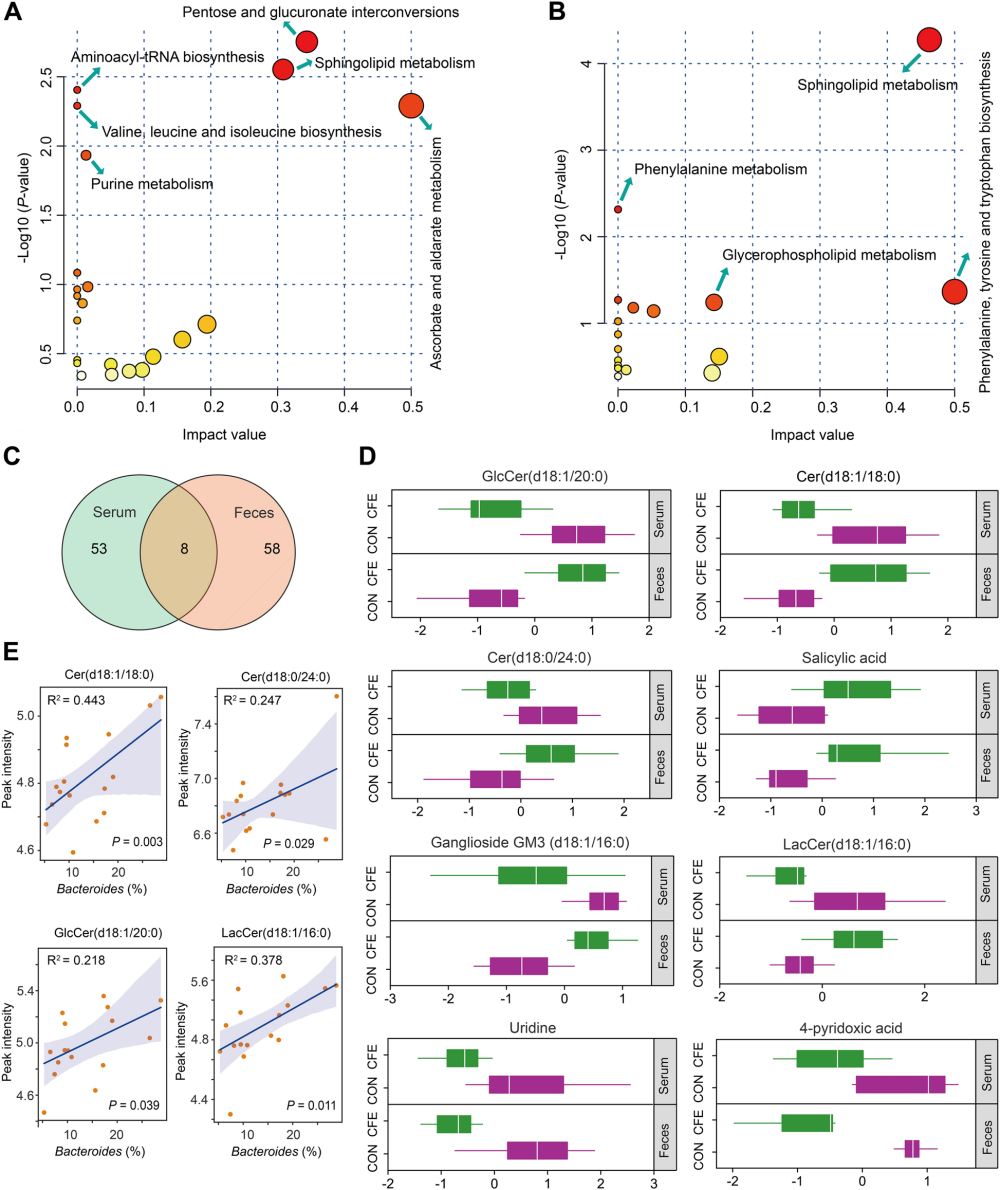
Fecal and serum metabolome of CON and CFE cows. Metabolic pathway analysis conducted with the differentially abundant fecal metabolites (**A**) and serum metabolites (**B**). **C** Venn diagram outlining the shared differentially abundant metabolites in fecal and serum samples. **D** Boxplots showing relative abundance of shared metabolites in serum and fecal samples between CON and CFE cows. Relative abundances of metabolites are visualized after normalization. **E** Regression lines showing the relationships between fecal *Bacteroides* and fecal Cer(d18:1/18:0), Cer(d18:0/24:0), GlcCer(d18:1/20:0), or LacCer(d18:1/16:0). CON, control; CFE, citrus flavonoid extract

Fecal metabolomics pathway analysis based on differential metabolites showed the enrichment of “pentose and glucuronate interconversions,” “sphingolipid metabolism,” “aminoacyl-tRNA biosynthesis,” “valine, leucine and isoleucine biosynthesis,” “ascorbate and aldarate metabolism,” and “purine metabolism” were the significantly different pathways (*P* < 0.05; Fig. 4A). For the serum metabolome, “sphingolipid metabolism,” phenylalanine metabolism,” and “phenylalanine, tyrosine and tryptophan biosynthesis” were the significantly different pathways (*P* < 0.05; Fig. 4B). Interestingly, we found that the common pathway enriched in comparisons of CFE vs. CON was “sphingolipid metabolism”. We also identified 8 shared differential metabolites in feces and serum, including GlcCer(d18:1/20:0), Cer(d18:1/18:0), Cer(d18:0/24:0), salicylic acid, ganglioside GM3(d18:1/16:0), LacCer(d18:1/16:0), uridine, and 4-pryidoxic acid (Fig. 4C and D), and four of these differential metabolites belonged to sphingolipids. Microbial species within Bacteroides are the only gut commensal bacteria known to synthesize sphingolipids [40]. Thus, we further evaluated the relationship between *Bacteroides* and significantly altered sphingolipid species in feces. As shown in Fig. 4E, GlcCer(d18:1/20:0), Cer(d18:1/18:0), Cer(d18:0/24:0), and LacCer(d18:1/16:0) were positively correlated with the relative abundance of Bacteroides.

To examine the potential relationship between changed hindgut bacteria and altered fecal or serum metabolites, the Spearman correlation coefficient was calculated between differential metabolites and the abundance of hindgut bacteria. Statistical significance (*P* < 0.05) and correlation coefficient (r|> 0.5) indicate the correlations. For fecal metabolites, negative correlations were found mainly between potentially beneficial microbes *Bacteroides* and *Bifidobacterium* and guanosine, 4-pyridoxic acid, digitoxigenin, methyl palmitate, 3,7,8,15-scirpenetetrol, 1,3,8-naphthalenertriol, homovanillin, (R)-( +)-pulegone, beta-lapachone, threoninyl-proline, barbital, and chenodeoxycholic acid (Fig. S1A). Most of downregulated fecal metabolites exhibited positive associations with *Escherichia-Shigella*, *Alloprevotella*, and *Paeniclostridium*. Compared with fecal metabolites, fewer associations existed between serum metabolites and fecal microbiota (Fig. S1B). We also observed that serum Cer(d18:1/18:0) and Cer(d18:0/24:0) were negatively correlated with the relative abundance of fecal *Bacteroides* (Fig. S2A).

To further examine the associations between fecal or serum metabolites and serum biochemical parameters, we selected eight common metabolites associated with serum parameters using Spearman’s correlation. Statistical significance (*P* < 0.05) and correlation coefficient (*r*|> 0.5) indicate the correlations. We found that serum sphingolipids LacCer(d18:1/16:0), ganglioside GM3 (d18:1/16:0), Cer(d18:1/18:0), and uridine were positively correlated with serum TNF-α, LBP, and LPS. Serum salicylic acid was negatively associated with serum LPS, TNF-α, and IL-6 (Fig. S2B). Serum GlcCer(d18:1/20:0) was positively correlated with serum LBP, Hp, and ALT. Fecal 4-pyridoxic acid was positively correlated with serum ALT, IL-6, and TNF-α. Fecal GlcCer(d18:1/20:0) was negatively correlated with serum ALT, Hp, and LPS. Compared with fecal metabolites, serum metabolites have more associations with serum parameters. These findings suggest that host metabolites are highly correlated with systemic inflammation and endotoxin.

### Alteration of the serum lipid profile

**Fig. 5:**
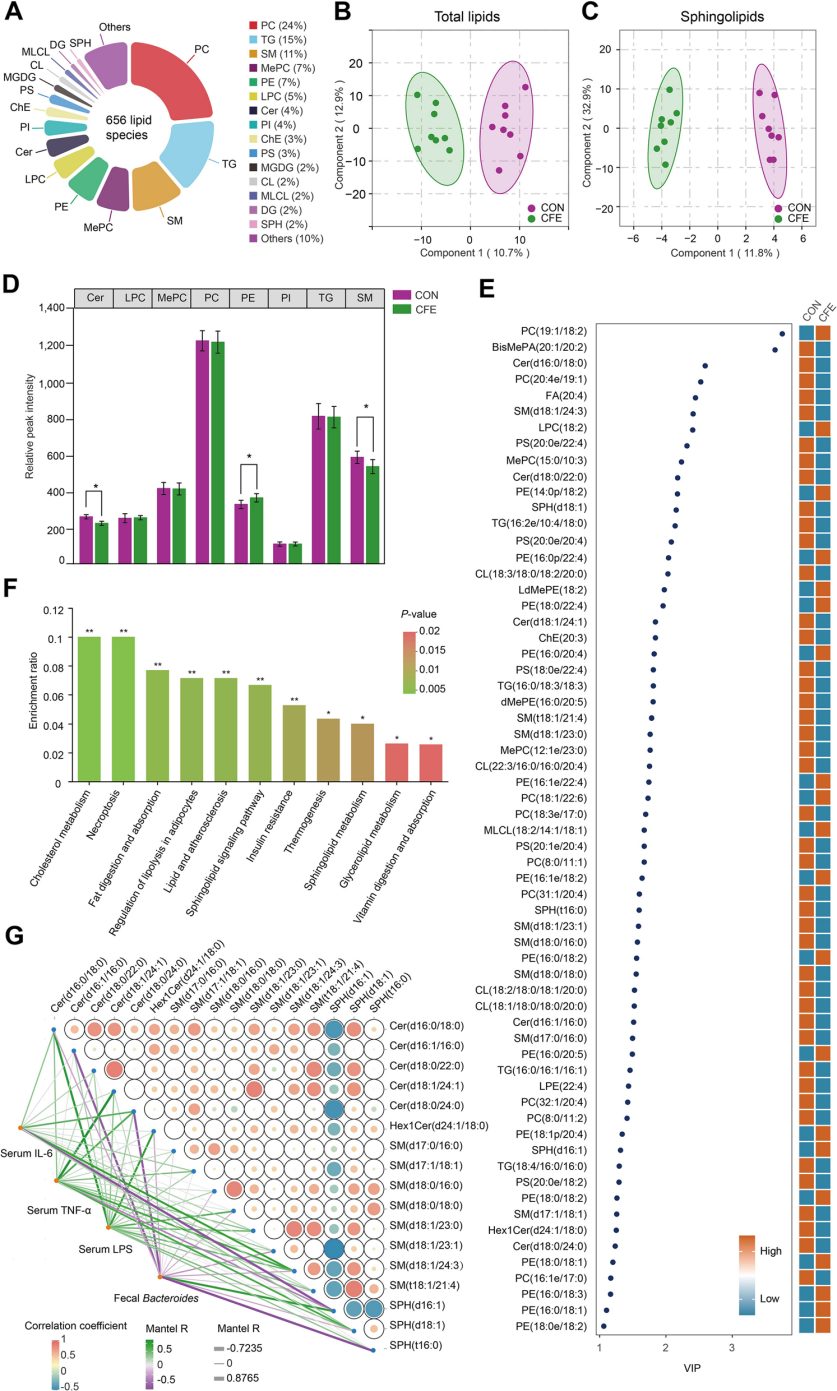
Serum lipidomics analysis. **A** Percentages of numbers of main lipid subclasses. **B** Principal component analysis (PCA) plot of total lipids. **C** PCA plot of serum sphingolipids (including SM, Cer, SPH). **D** Comparison of the contents of top eight lipid subclasses between CFE vs. CON. *P < 0.05. **E** Heatmap of differentially abundant serum lipids (VIP > 1, *P* < 0.05 and |FC|≥ 1.2). **F** KEGG enrichment analysis based on differentially abundant serum lipids. *P < 0.05, **P < 0.01. **G** The heatmap on the right depicts the correlations between differential serum sphingolipid species. Red, positive correlations; bule, negative correlations. The heatmap on the left depicts the correlations of serum IL-6, TNF-α, LPS, fecal *Bacteroides* with differential serum sphingolipid species. Green, positive correlations; purple, negative correlations. CON, control; CFE, citrus flavonoid extract; VIP, variable importance in projection; FC, fold change; LPS, lipopolysaccharide

Given the suggested association between several sphingolipid species and hindgut metabolism, we further assessed the effects of dietary CFE on the serum lipidome of dairy cows. A total of 656 lipid molecules were identified in positive and negative ion modes. At the subclass level, the major lipid species were phosphatidylcholine (PC, 25%), triglyceride (TG, 15%), sphingomyelin (SM, 11%), methanol-phosphatidylcholine (MePC, 7%), phosphatidylethanolamine (PE, 7%), lysophosphatidyl choline (LPC, 11.07%), and Cer (4%) (Fig. 5A). As determined by PCA analysis, CFE caused a significant lipid metabolic perturbation in serum (Fig. 5B). The most abundantly identified lipid subclasses belonging to sphingolipids are SM, Cer, sphinganine (SPH), Hex1Cer, and Hex2Cer. We also analyzed the difference in sphingolipids between CON and CFE. For sphingolipids, the PCA plot showed a discrimination between CON and CFE (Fig. 5C), which indicated a significant change in serum sphingolipids after CFE treatment. The total contents of the top eight lipid subclasses were compared between CFE and CON. As shown in Fig. 5D, compared with CON, the CFE dairy cows had significantly lower concentrations of serum Cer (*P* = 0.032) and SM (*P* = 0.024). The serum PE concentration in CFE cows was significantly higher (*P* = 0.028) than that in the CON cows.

As shown in Fig. 5E, 63 out of 656 serum lipid species were significantly altered (*P* < 0.05). Twenty-one features were found to have significantly (*P* < 0.05) higher concentrations in CFE [e.g., PC(19:1/18:2), PE(16:0p/22:4), and SPH(d18:1)] and 42 were more abundant (*P* < 0.05) in the serum of CON [e.g., BisMePA(20:1/20:2), Cer(d16:0/18:0), and SM(d18:1/24:3)]. Additionally, 11 enriched pathways with the most significant differences between CON and CFE were identified, including “cholesterol metabolism,” “necroptosis,” “fat digestion and absorption,” “regulation of lipolysis in adipocytes,” “lipid and atherosclerosis,” “sphingolipid signaling pathway,” “insulin resistance,” “thermogenesis,” “sphingolipid metabolism,” “glycerolipid metabolism,” and “vitamin digestion and absorption” (Fig. 5F). Furthermore, Spearman’s correlation analysis was performed to investigate the correlation between differentially abundant lipid species and the correlation between the differentially abundant lipid species and serum IL-6, TNF-α, LPS, and fecal *Bacteroides* (*P* < 0.05 and the correlation coefficient >|0.6|) (Fig. 5G). The dynamic network heatmap showed that Cer(d16:0/18:0), Cer (d18:0/22:0), and Cer(d18:1/24:1) were positively correlated with SPH(d18:1), SM(t18:1/21:4), SM(d18:1/23:0), and Cer(d18:1/24:1). SM(d18:0/16:0) was positively correlated with SPH(t16:0), SPH(d18:1), and SM(d18:0/18:0). SPH(d16:1) was negatively correlated with Cer(d16:0/18:0), Cer(d18:0/22:0), Cer(d18:0/24:0), SM(d18:1/23:1), SM(d18:1/24:3), and SM(t18:1/21:1). Serum IL-6 was positively correlated with SM(d18:1/24:3) and negatively correlated with SPH(d16:1). Serum TNF-α was positively correlated with Cer(d18:1/24:1). Serum LPS was positively correlated Cer(d16:0/18:0) and Hex1Cer(d24:1/18:0). Fecal *Bacteroides* was negatively correlated with Cer(d16:1/16:0), Cer(d18:0/24:0), and SPH(t16:0).

### Changes in fecal metaproteomics profiling

Metaproteomics allows us to understand the functional role of microbiota and their interactions with the host in an ecosystem. Thus, the metaproteomics approach was used to determine hindgut bacterial protein expression. Before investigating the proteins detected by the high-throughput experiment, the quality of the mass spectrometry data was evaluated. The length distribution of peptide segments (Fig. S3A) reveals that the majority of detected peptide segments fall within the range of 8–32. More than half of the proteins had only 1–2 peptides (Fig. S3B). Approximately 98% of the proteins had a mass > 10 kDa, which indicates very good coverage (Fig. S3C). Sequence coverage distributions greater than 10 and 20% were 42.7 and 21.9% respectively (Fig. S3D). We detected 952,359 total spectra, 59,447 identified spectra, 9295 peptides, and 4493 protein groups (Fig. S3E).

**Fig. 6:**
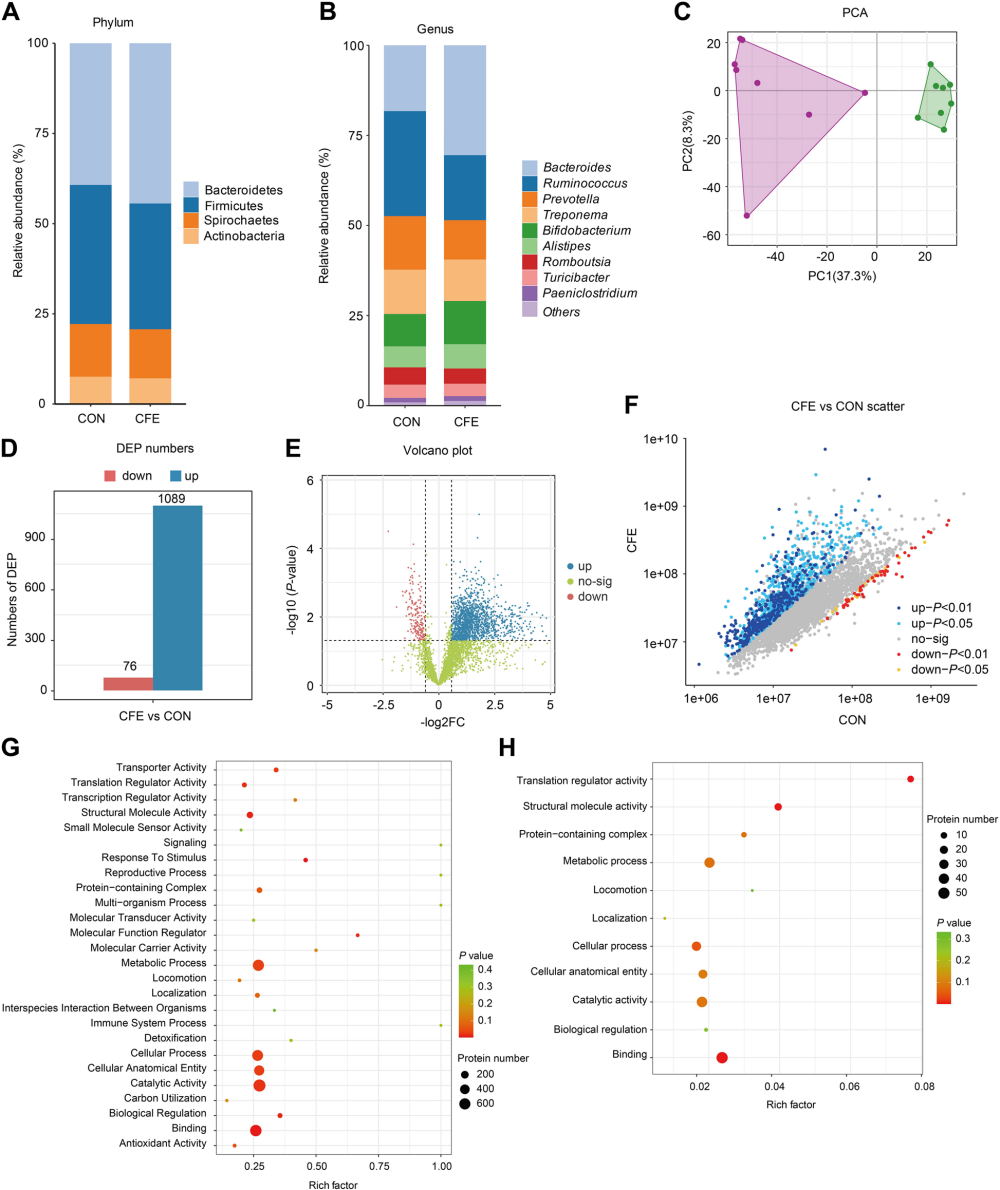
Dietary CFE induced fecal metaproteomics alterations. Taxonomic composition of fecal samples according to their metaproteomic profile at the phylum level (**A**) and genus level (**B**). **C** Principal component analysis (PCA) plot of fecal metaproteomics. **D** Differentially expressed proteins (DEP) numbers. **E** Volcano plot of DEP. **F** Scatter plot of DEP. Analysis of GO enrichment for upregulated DEP (**G**) and downregulated DEP

Taxonomic analysis was conducted using metaproteomics, and metabolically active taxa of the microbiota were identified. At the phylum level, Bacteroidetes, Firmicutes, Spirochaetes, and Actinobacteria were observed as the dominant phyla (Fig. 6A). The dominant genera in feces were *Bacteroides*, *Ruminococcus*, *Prevotella*, *Treponema*, and *Bifidobacterium* which comprised approximately 70% of average relative abundance (Fig. 6B). The PCA plot showed a complete separation between CON and CFE (Fig. 6C), indicating that dietary CFE caused significant changes in hindgut bacterial protein in the dairy cows. The resulting plots showed the DEP, including 1089 upregulated DEP and 76 downregulated DEP (Fig. 6D–F). To better understand the function of 1165 DEP, GO enrichment analysis was used to do further analysis. For upregulated DEP, “binding,” “catalytic activity,” “cellular anatomical entity,” “cellular process,” and “metabolic process” were significantly enriched. “Structural molecule activity,” “translation regulator activity,” and “binding” were significantly enriched according to downregulated DEP. The KEGG enrichment analysis showed that “ribosome,” “biosynthesis of amino acids,” “carbon metabolism,” “biosynthesis of antibiotics,” “carbon fixation pathways in prokaryotes,” “carbon fixation in photosynthetic organisms,” “methane metabolism,” “butanoate metabolism,” “pyruvate metabolism,” “purine metabolism,” “fructose and mannose metabolism,” and “glycolysis/ gluconeogenesis,” were the main enriched pathways based on upregulated proteins (Fig. S4A). The metabolic pathways “ribosome,” “biosynthesis of amino acids,” “carbon metabolism,” “biosynthesis of antibiotics,” “carbon fixation pathways in prokaryotes,” and “glycolysis/gluconeogenesis” were significantly enriched according to downregulated proteins (Fig. S4B).

**Fig. 7:**
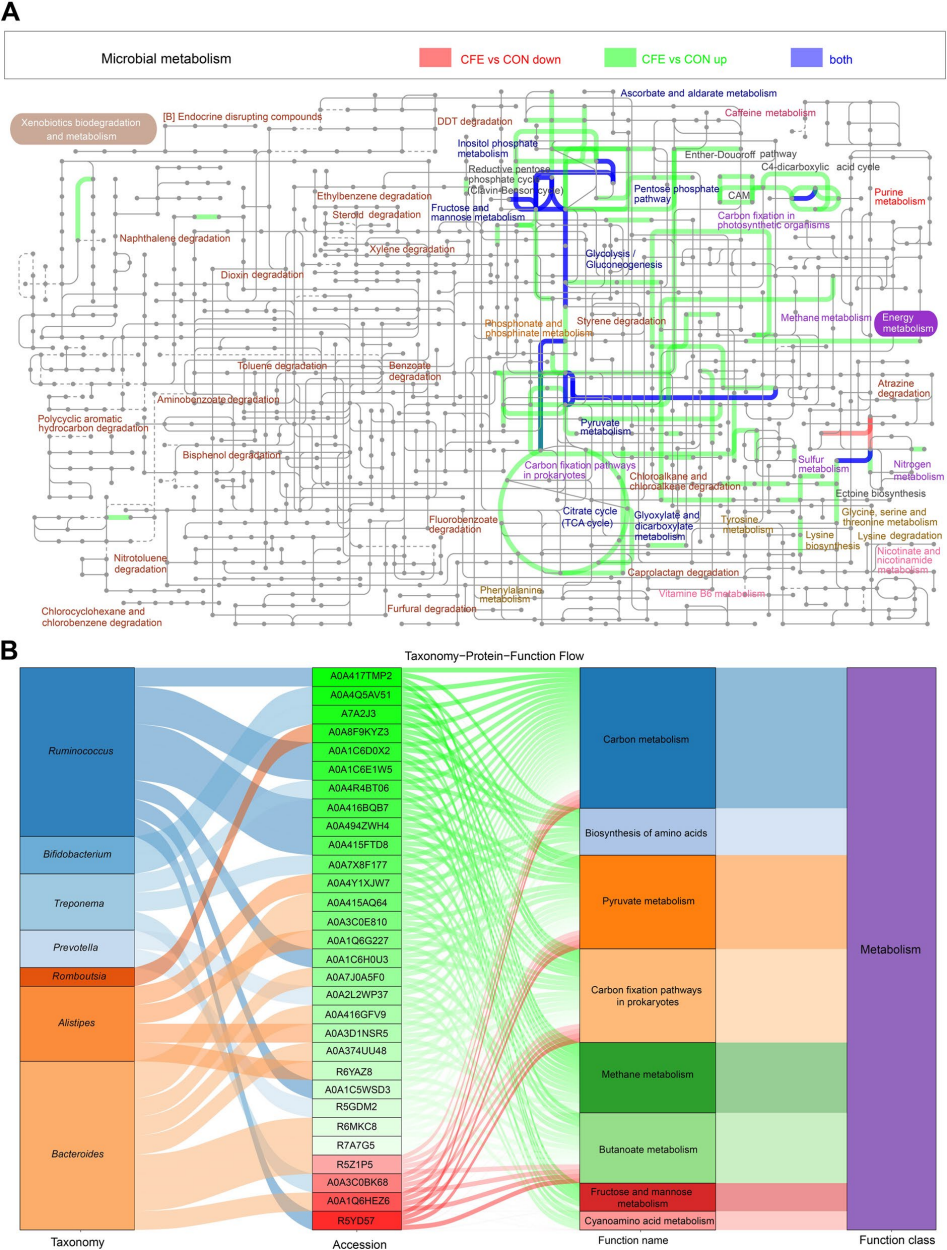
Function analysis of differentially expressed proteins (DEP). **A** Interactive Pathways Explorer (iPath) analysis of microbial metabolism. Green: increase; red: decrease. **B** Sankey diagram of taxonomy-DEP-function flow

**Fig. 8:**
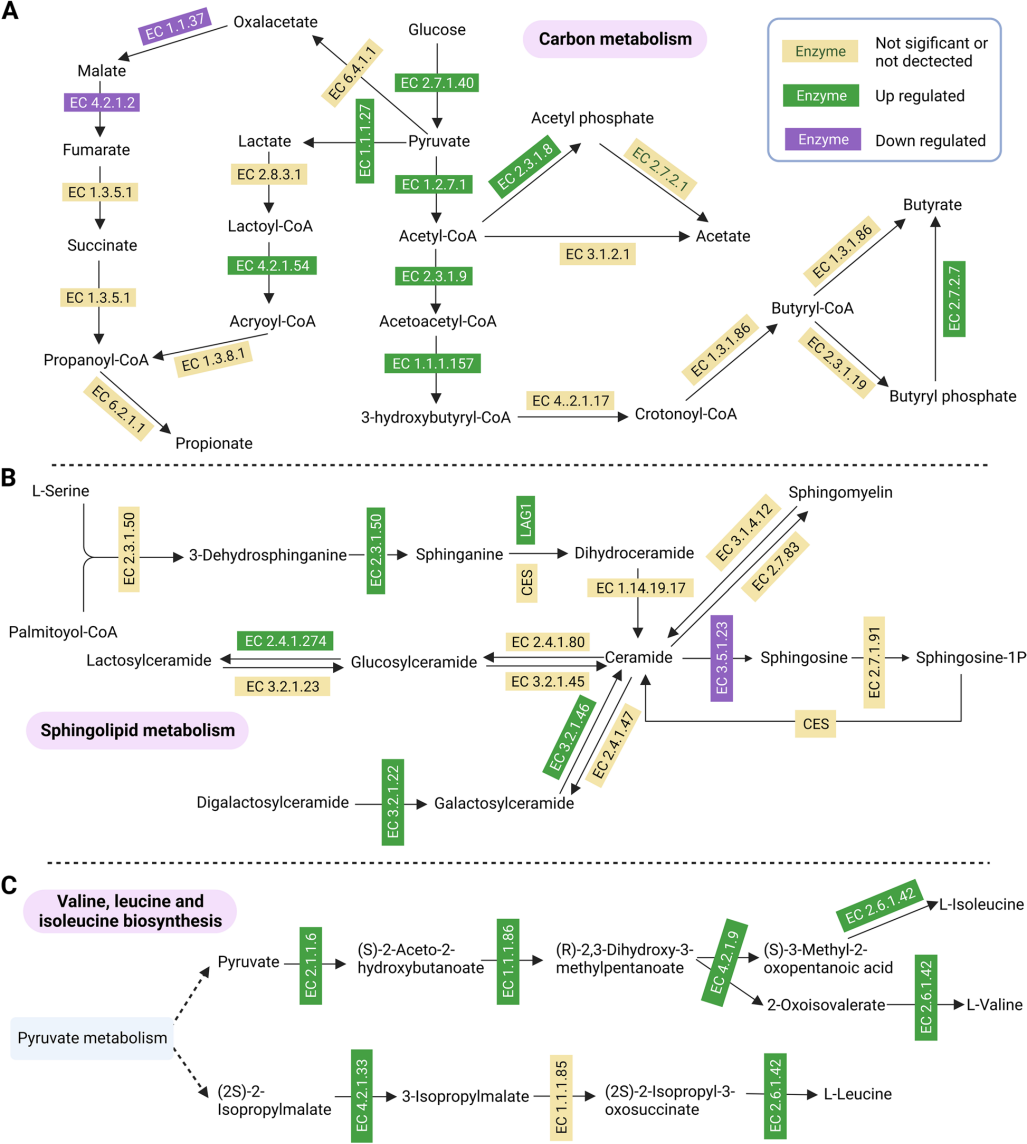
Metabolic pathways associated with differential abundant fecal metabolites. **A** Metabolic pathways for short-chain fatty acid and methane production by microbial conversion from carbohydrates. **B** Metabolic pathways for ceramide biosynthesis and conversion. **C** Metabolic pathways for valine, leucine, and isoleucine biosynthesis. Differentially expressed proteins (enzymes) in the pathways are shown in green (increase in CFE cows) and purple (decrease in CFE cows)

iPath was also used to compare the metaproteomes of CON and CFE. The results are summarized in an overview map, on which green lines show upregulated pathways, and red lines show downregulated pathways. We observed that the downregulated pathways focused on carbohydrate metabolism and energy metabolism. The Sankey diagram showed the degree of connection among taxonomy, DEP, and KEGG function (Fig. 7B). The results indicated that the differential genera *Bacteroides*, *Alistipes*, and *Bifidobacterium* played important roles in carbohydrate metabolism and energy metabolism. In the present study, fecal butyrate, GlcCer(d18:1/20:0), Cer(d18:1/18:0), Cer(d18:0/24:0), and LacCer(d18:1/16:0), leucine, and isoleucine were different between two treatments. To determine the effect of the CFE on microbial functions, the proteins involved in “carbon metabolism,” “sphingolipid metabolism,” and “valine, leucine and isoleucine biosynthesis” were closely examined using metaproteomic data. We found that some key enzymes including EC 2.3.1.9, EC 1.1.1.157, and EC2.7.2.7 were enriched in butanoate metabolism pathway in CFE cows (Fig. 8A). The enrichment of EC2.3.1.50 and LAG1 could contribute to the increase of Cer in the CFE cows (Fig. 8B). For the pathway “valine, leucine and isoleucine biosynthesis,” the main enzymes including EC 2.1.1.6 and EC 4.2.1.33 were enriched in the CFE cows (Fig. 8C).

## Discussion

In dairy cows, optimal performance requires feeding of “higher-energy” diets, i.e., diets in which the high content of starch favors the production of the main glucogenic precursor, propionate. A meta-analysis showed that feeding cattle more than 44.1% concentrate or less than 39.2% neutral detergent fiber in the diet was associated with gastrointestinal dysbiosis and an increase in the risk of systemic inflammation [8]. Gastrointestinal dysbiosis changes the composition and reduces the functionality of rumen and hindgut microbiota by promoting the proliferation of opportunistic bacteria and disintegration and lysis of those that cannot adapt to suboptimal rumen or hindgut environments (e.g., pH; [41]). As a result, endotoxins, such as LPS from gram-negative bacteria, are released into the ruminal and intestinal digesta [41]. These releases increase the concentration of luminal endotoxins and contribute to gut epithelial damage [42] and rumen papillae [43]. Available data indicate that gastrointestinal tract-derived LPS increases the concentrations of acute phase proteins (e.g., SAA and Hp) and the inflammatory cytokines (e.g., IL-1β and TNF-α) in the blood by regulating a large number of immune genes, eventually leading to a state of systemic inflammation [44]. Inflammation originating from the translocation of LPS markedly shifts nutrient partitioning toward the activated immune system and away from productive phenotypes.

Previous studies demonstrated that feeding starch-rich diets increased systemic concentrations of endotoxin and inflammatory biomarkers by damaging gastrointestinal function [44–46]. The study of the ruminal ecosystem has expanded over the past two decades, with particular attention being paid to regulating the rumen microbiome in response to increasing rapidly fermentable carbohydrates [25, 47]. Our own unpublished data showed that feeding CFE did not alter rumen fermentation parameters, ruminal LPS, or ruminal microbial diversity in mid-lactation dairy cows fed the high-starch diet. However, supplementation with CFE reduced the relative abundances of the genera *Shuttleworthia* and *Olsenella*, which are known starch utilizers [48]. Microbes and digesta metabolites and their effects on the function and expression of genes of the lower gut are not well characterized, thus generating the need for further research. In the present study, supplementing CFE decreased fecal LPS. Several indirect metrics of gut health, including acute phase proteins (Hp, LBP), inflammatory cytokines (IL-6, TNF-α), and serum LPS concentrations, were inhibited by supplementing CFE. The lower fecal LPS could cause the reduction in circulating LPS, which partly explains the decrease in acute phase proteins and inflammatory cytokines. These results suggested that CFE attenuated the high-grain feeding-induced inflammation by reducing the systemic LPS level and inhibiting LPS-activated inflammatory response. Additionally, in the liver, LPS causes hepatic inflammation and hepatocyte injury, suppressing liver function [49]. Previous studies by some authors have revealed a drastic change in inflammatory status in the liver due to high-grain feeding [50, 51]. ALT and AST are released into the blood when hepatocellular damage or death occurs [52]. The CFE cows had lower ALT concentrations, which indicated that CFE had the potential of alleviating liver injury.

The role of specific dietary phytochemicals has been robustly linked in recent years to changes in gut microbial flora. In non-ruminants, emerging evidence suggests that in vivo metabolic processes, particularly transformation by gut microbiota, may elicit or enhance the intrinsic bioactivities of citrus flavonoids, and have a positive impact on host health [53, 54]. Previous studies have demonstrated that high-starch feeding could cause the disturbance of the hindgut microbial community in dairy cows [55, 56]. Citrus flavonoids, including naringin and hesperidin, are well-known antibacterial plant secondary metabolites, because they can break membranes, prevent the synthesis of nucleic acids, kill, or suppress bacterial cells, and reduce bacterial pathogenicity [57, 58]. In the present study, the β-diversity analysis of the fecal microbiota revealed that the CFE supplementation resulted in significant alterations in its overall composition; however, there were no statistically significant differences between the α-diversity indices of the samples. Therefore, this may imply that feeding CFE resulted in selective changes in the hindgut microbial community structure, but there were no more global changes in richness and evenness. Citrus flavonoids have been shown to inhibit the growth and adhesion of gut pathogens to a human gut cell line, and to enhance the proliferation and adhesion of probiotics [59]. Our study showed that dietary CFE increased the relative abundance of *Bacteroides*, *Bifidobacterium*, *Alistipes*, *Akkermansia*, and *Blautia* in the hindgut of dairy cows. Bacteria classified in the genera *Bifidobacterium* and *Akkermansia* are universally acknowledged as important probiotics [57, 58]. *Blautia* is a new functional genus defined as having potential probiotic properties. This was attributed to the contribution of *Blautia* in reducing inflammation and metabolic diseases as well as its antimicrobial activity against specific microorganisms [60]. These beneficial genera were highly abundant in the CFE cows, which suggested that citrus flavonoids could exert a prebiotic effect. The opportunistic pathogen *Escherichia-Shigella* was inhibited by CFE. The high abundance of *Escherichia-Shigella* in the gut microbiome may be linked to intestinal inflammation [61]. Thus, the results suggested that CFE can modify the community of the hindgut microbiota by increasing the growth of beneficial bacteria and limiting the growth of harmful bacteria, consequently exhibiting health-promoting effects on dairy cows fed a high-starch diet.

Most gut bacteria exert their effects through the generation of secondary metabolites via various metabolic pathways, including SCFAs, vitamins, and amino acids. Butyrate has positive effects on regulating intestinal immune function and the inflammatory response [62]. We observed that CFE increased the production of butyrate. This observation suggests stimulation of the butyryl CoA-acetyl CoA transferase pathway [63], and associated proteins were enriched in CFE cows according to fecal metaproteomic analysis in our study. Therefore, it can be inferred that CFE may enhance hindgut barrier function by stimulating butyrate production.

Generally, the altered composition of the gut microbiota might cause changes in microbial metabolites which might further affect the host whole metabolome. We observed that 4-pyridoxic acid, salicylic acid, and uridine were common differentially abundant metabolites between fecal and serum samples. 4-Pyridoxic acid is the principal catabolite of pyridoxal and is linked to systemic inflammation [64]. Zhang et al. [65] demonstrated that salicylic acid negatively affects the prevalence of *Prevotella*, hence inhibiting the inflammatory response. Furthermore, 4-pyridoxic acid and salicylic acid were regarded as prediction factors of left displaced abomasum in dairy cows [34]. In this study, changes in the levels of these two metabolites may be linked with decreased systemic inflammation induced by CFE supplementation. The branched-chain amino acids (BCAA) leucine and isoleucine were increased in the feces of CFE cows. BCAA are synthesized by the gut microbiota and play a critical role in maintaining homeostasis by regulating glucose and lipid metabolism [66]. Supplementation of mice with a mixture of BCAA promotes a healthy microbiota with an increase in *Akkermansia* and *Bifidobacterium* [67]. Elevated fecal BCAA levels could be associated with the increase of *Akkermansia* and *Bifidobacterium* in our study. Intestinal inflammation is linked to aminoacyl-tRNA biosynthesis, which entails active amino acids attaching to tRNA through an ester bond to generate the corresponding aminoacyl-tRNA [68]. Ma et al. [68] demonstrated a positive correlation between the relative abundance of *Akkermansia* and the biomarker for aminoacyl-tRNA biosynthesis. TNF-α and IL-6 expression were inversely associated with aminoacyl-tRNA biosynthesis [69]. Our study indicated that CFE could increase the abundance of *Akkermansia* and decrease the levels of TNF-α and IL-6, indicating that CFE might upregulate aminoacyl-tRNA biosynthesis against hindgut inflammation.

This study observed significant changes in the hindgut and host metabolic pathways after CFE treatment. Significantly, these alterations were mainly manifested in sphingolipid metabolism. The enzyme serine palmitoyltransferase (SPT), which catalyzes the interaction between serine and an acyl-CoA thioester (such as palmitoyl-CoA), forms 3-ketosphinganine and is necessary to produce sphingolipids. This SPT-mediated process is the committed step in sphingolipid production in bacterial and eukaryotic cells [70]. For CFE-fed dairy cows, the increase in fecal ceramides was associated with the increase in enzymes involved in synthesizing ceramides, suggesting an accelerated turnover of ceramides in the hindgut. Most Bacteroidetes and certain alpha-Proteobacteria species synthesize sphingolipids, but Bacteroidetes are the only gut commensal bacteria that synthesize sphingolipids [40]. Thus, the increased abundance of Bacteroides could contribute to the enhancement of hindgut GlcCer(d18:1/20:0), Cer(d18:1/18:0), Cer(d18:0/24:0), and LacCer(d18:1/16:0). Interestingly, we found that these four sphingolipid species were downregulated in the serum of CFE cows. In mouse models, Bacteroides-derived ceramide can enter host metabolic pathways and affect host lipid homeostasis [70]. De novo sphingolipid synthesis in hosts is inversely proportional to microbial sphingolipid synthesis [71], indicating that hosts may be able to sense sphingolipid levels and alter production accordingly. According to Formes et al. [72], in the absence of microbiota, the liver endothelium boosts the host’s capability of endogenous sphingosine production to guarantee sphingosine-1-phosphate-signaling. Our results may suggest that CFE addition increased Bacteroides-derived sphingolipids, which are critical for improving hindgut homeostasis, and sphingolipids produced by the hindgut microbiota have the potential to pass the epithelial barrier and interact with dairy cow metabolism.

Although gut-derived ceramides play an essential role in maintaining intestinal homeostasis, excessive accumulation of host ceramides may cause many metabolic diseases, such as important mediators in metabolic diseases [73, 74]. The correlation analysis conducted in the present study revealed a close relationship between inflammatory cytokines and serum ceramide. On the one hand, inflammatory cytokines, such as TNF-α and interleukins, stimulate the production of host ceramide [75]. Cytokines induce ceramide formation by promoting the expression of genes involved in ceramide biosynthesis (e.g., SPT) and by enhancing the expression and activity of sphingomyelin hydrolyzing enzymes [76]. In addition, a synergistic effect of saturated free fatty acids and LPS can stimulate ceramide synthesis [77, 78]. On the other hand, Vandanmagsar et al. [79] confirmed that ceramides themselves may trigger inflammation by activating the NLR family pyrin domain containing 3 and boosting the release of IL-1β and IL-18. In the present study, CFE supplementation for dairy cows significantly reduced serum ceramide concentrations based on lipidomic analysis. Several ceramide species in serum correlated with fecal *Bacteroides*, serum proinflammatory factors, and endotoxin in dairy cows, accounting for the improvements in systemic inflammation. Phytonutrients from plant-based diets especially polyphenols of both flavonoid and non-flavonoid sources could potentially reduce the specific ceramides in metabolic syndrome such as type II diabetes and obesity in non-ruminant models [80, 81]. Our results indicated that alleviating gut dysbiosis have significant impacts on host homeostasis via the regulation of gut sphingolipid metabolism using plant flavonoids in dairy cows. However, the exact mechanism of citrus flavonoids involved in ceramide metabolism modulation still needs to be understood at the molecular level to gain a better understanding of their mechanism of action. Furthermore, the cows used herein were in mid-lactation because the mid-lactation period is relatively stable, and the gastrointestinal microbial communities have adapted to the increase in dry matter intake and high-grain feeding during early lactation. Considering that the vast majority of diseases [82] and host ceramide accrual [83] in dairy cattle occur during early lactation, future studies are warranted to further examine the effects of CFE on the hindgut microbiome and host homeostasis in early-lactating cows.

## Conclusions

**Fig. 9:**
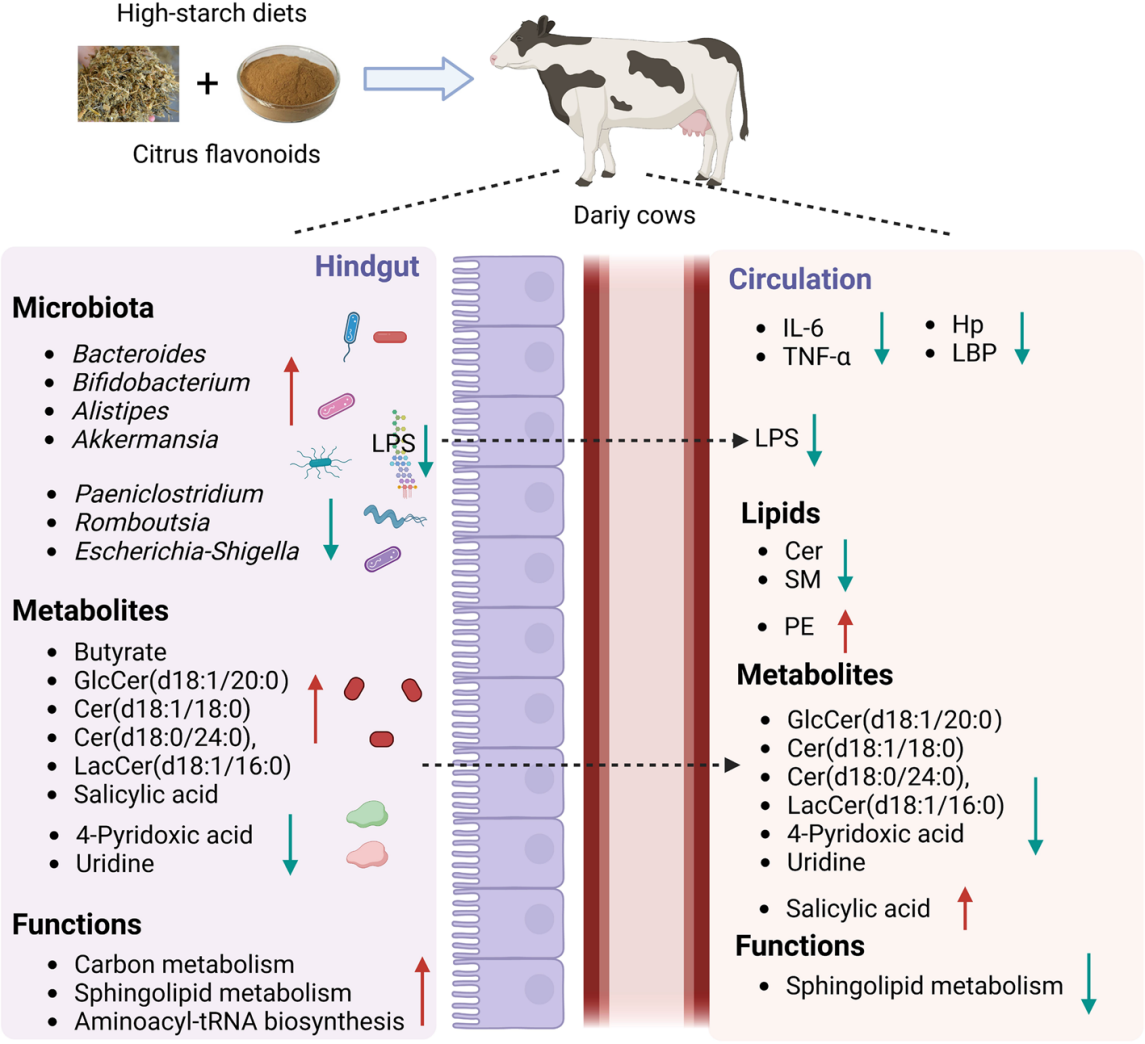
A summary of the findings and proposed mechanism of dietary citrus flavonoids on improving hindgut microbiota and host homeostasis in dairy cows consuming a high-starch diet. Hp, haptoglobin; LPS, lipopolysaccharide; LBP, LPS-binding protein; Cer, ceramide; SM, sphingomyelin; PE, phosphatidylethanolamine

Our results showed that citrus flavonoids decreased the levels of inflammatory cytokines, fecal LPS, and serum LPS in dairy cows fed a high-starch diet, and the improvement in metabolic homeostasis was linked to the modulation of hindgut microbial metabolism (Fig. 9). Dietary CFE altered the structure and composition of the hindgut microbiota by promoting beneficial bacterial growth and correspondingly inhibiting harmful bacteria. We reported that microbial biosynthesis of ceramides and fecal *Bacteroides* were both increased in cows receiving CFE. In contrast, the dietary CFE reduced host ceramide biosynthesis, which was associated with inflammatory cytokines. In addition, the strengthened metabolic functions of gut microbiota, including microbial sphingolipid metabolism and carbohydrate metabolism, were proven by metaproteomics. Our study revealed potential links between the hindgut microbiome, sphingolipid metabolism, and systemic inflammation in dairy cows. The results will provide a clue to the better understanding of the contribution of bacterial sphingolipids to host metabolism and their potential role as host homeostasis regulators in ruminants. Together, using plant flavonoids could be considered as a nutritional strategy for decreasing the risk of systemic inflammation in dairy cows fed a high-starch diet. Modulation of ceramide synthesis pathways by natural flavonoids may thus attract significant interest as bioactive agents in the maintenance of immunometabolic homeostasis of dairy cows with gut dysbiosis. The exact mechanism of flavonoids involved in ceramide modulation needs to be understood at the molecular level to gain more insights into their mechanistic mode of action in ruminants.

### Supplementary Information

**Additional file 1: Table S1.** Chemical composition of the citrus flavonoid extract. **Table S2.** Ingredient and chemical composition of the basal lactation TMR. **Table S3.** Effects of dietary supplementation of citrus flavonoid extract on the main fecal microbiota (> 0.05% at the phylum level) in dairy cows.

**Additional file 2: Fig. S1.** Heatmap of association analysis between significantly altered fecal bacteria and differential metabolites in feces (A) and serum (B). Only features showing strong significant correlations (|r| > 0.5 and *P* < 0.05) were visualized. **Fig. S2.** Association analysis among fecal bacteria, differential metabolites, and serum biochemical parameters. (A) Regression lines showing the relationships between fecal Bacteroides and serum Cer(d18:1/18:0) and Cer(d18:0/24:0). (B) Heatmap of association between shared metabolites and serum biochemical parameters. Only features showing strong significant correlations (|r| > 0.5 and *P* < 0.05) were visualized. ALT, alanine aminotransferase; Hp, haptoglobin; LBP, LPS-binding protein; LPS, lipopolysaccharide. **Fig. S3.** Identification and quantitative evaluation of identified fecal proteins. (A) Distribution of proteins based on peptide length distribution. (B) Distribution of proteins based on the number of peptides. (C) Distribution of proteins based on molecular weight. (D) Distribution of identified protein sequences. (E) Protein information. **Fig. S4**. KEGG enrichment analysis of differentially expressed proteins (DEM). (A) Upregulated DEM. (B) Downregulated DEM.

## Data Availability

The raw sequence data reported in this paper have been deposited in the Genome Sequence Archive in National Genomics Data Center, China National Center for Bioinformation / Beijing Institute of Genomics, Chinese Academy of Sciences (GSA: CRA009531) that are publicly accessible at https://ngdc.cncb.ac.cn/gsa.
