# Supplementary material for: Integrated multi-omics analysis reveals the positive leverage of citrus flavonoids on hindgut microbiota and host homeostasis by modulating sphingolipid metabolism in mid-lactation dairy cows consuming a high-starch diet
Source: Microbiome. 2023 Oct 25;11:236. doi: 10.1186/s40168-023-01661-4 (PMC10598921; doi:10.1186/s40168-023-01661-4)
Supplement: Supplementary file 2 — Additional file 1: Table S1. Chemical composition of the citrus flavonoid extract. Table S2. Ingredient and chemical composition of the basal lactation TMR. Table S3. Effects of dietary supplementation of citrus flavonoid extract on the main fecal microbiota (> 0.05% at the phylum level) in dairy cows. [file 40168_2023_1661_MOESM1_ESM.docx]

Table S1. Chemical composition of the citrus flavonoid extract.

| Item | Contents |
| --- | --- |
| Chemical composition, % of DM |  |
| DM, % as fed | 92.16 |
| OM | 85.37 |
| Total flavonoids | 56.83 |
| Naringin | 19.09 |
| Hesperidin | 13.34 |
| Neohesperidin | 7.73 |
| Nobiletin | 3.21 |
| Tangeretin | 2.07 |

DM = dry matter; OM = organic matter.

Table S2. Ingredient and chemical composition of the basal lactation TMR.

| Item^1^ | Contents |
| --- | --- |
| Ingredient, % of DM | |
| Corn silage | 28.18 |
| Alfalfa silage | 7.57 |
| Alfalfa hay | 4.10 |
| Ground corn grain | 35.71 |
| Soybean meal | 9.56 |
| Canola meal | 2.76 |
| Sugar beet pulp | 6.34 |
| Whole cottonseed | 2.62 |
| Corn gluten meal | 0.83 |
| Mineral and vitamin mix^2^ | 2.33 |
| Chemical composition, % of DM^3^ |  |
| DM, % as fed | 53.19 |
| OM | 92.36 |
| CP | 16.84 |
| NDF | 27.85 |
| Starch | 32.97 |
| EE | 4.15 |
| NE_L_ (Mcal/kg of DM)^4^ | 1.67 |

^1^DM, dry matter; OM, organic matter; CP, crude protein; NDF, neutral detergent fiber; EE, ether extract; NE_L_, Net energy for lactation.

^2^The premix contained vitamin A, 1,900 kU/kg; vitamin D, 250 kU/kg; vitamin E, 3,000 mg/kg; niacin, 4,000 mg/kg; Cu, 1,200 mg/kg; Fe, 525 mg/kg; Zn, 13,000 mg/kg; Mn, 5,500 mg/kg; I, 170 mg/kg; Co, 50 mg/kg; Se, 27 mg/kg.

^3^Analyzed values.

^4^Calculated according to NRC (2001).

| Taxon | Mean abundance (%) in cows that received^1^: | | SEM | *P*-value |
| --- | --- | --- | --- | --- |
|  | CON | CFE |  |  |
| Firmicutes | 60.10 | 58.33 | 2.973 | 0.735 |
| Bacteroidetes | 28.67 | 36.25 | 3.308 | 0.642 |
| Actinobacteria | 8.42 | 4.13 | 1.991 | 0.566 |
| Spirochaetota | 1.61 | 0.62 | 0.404 | 0.208 |
| Patescibacteria | 0.85 | 0.41 | 0.235 | 0.198 |
| Proteobacteria | 0.20 | 0.12 | 0.091 | 0.613 |
| Cyanobacteria | 0.05 | 0.06 | 0.028 | 0.471 |
| Other | 0.10 | 0.08 | 0.037 | 0.725 |

Table S3. Effects of dietary supplementation of citrus flavonoid extract on the main fecal microbiota (> 0.05% at the phylum level) in dairy cows.

^1^CON, control; CFE, citrus flavonoid extract.
