# Supplementary material for: Integrated multi-omics analysis reveals the positive leverage of citrus flavonoids on hindgut microbiota and host homeostasis by modulating sphingolipid metabolism in mid-lactation dairy cows consuming a high-starch diet
Source: Microbiome. 2023 Oct 25;11:236. doi: 10.1186/s40168-023-01661-4 (PMC10598921; doi:10.1186/s40168-023-01661-4)

**Supplementary information**

**Fig. S1.** Heatmap of association analysis between significantly altered fecal bacteria and differential metabolites in feces (A) and serum (B). Only features showing strong significant correlations (|r| > 0.5 and *P* < 0.05) were visualized.

**Fig. S2.** Association analysis among fecal bacteria, differential metabolites, and serum biochemical parameters. (A) Regression lines showing the relationships between fecal *Bacteroides* and serum Cer(d18:1/18:0) and Cer(d18:0/24:0). (B) Heatmap of association between shared metabolites and serum biochemical parameters. Only features showing strong significant correlations (|r| > 0.5 and *P* < 0.05) were visualized. ALT, alanine aminotransferase; Hp, haptoglobin; LBP, LPS-binding protein; LPS, lipopolysaccharide.

**Fig. S3**. Identification and quantitative evaluation of identified fecal proteins. (A) Distribution of proteins based on peptide length distribution. (B) Distribution of proteins based on the number of peptides. (C) Distribution of of proteins based on molecular weight. (D) Distribution of identified protein sequences. (E) Protein information.

**Fig. S4.** KEGG enrichment analysis of differentially expressed proteins (DEM). (A) Up-regulated DEM. (B) Down-regulated DEM.


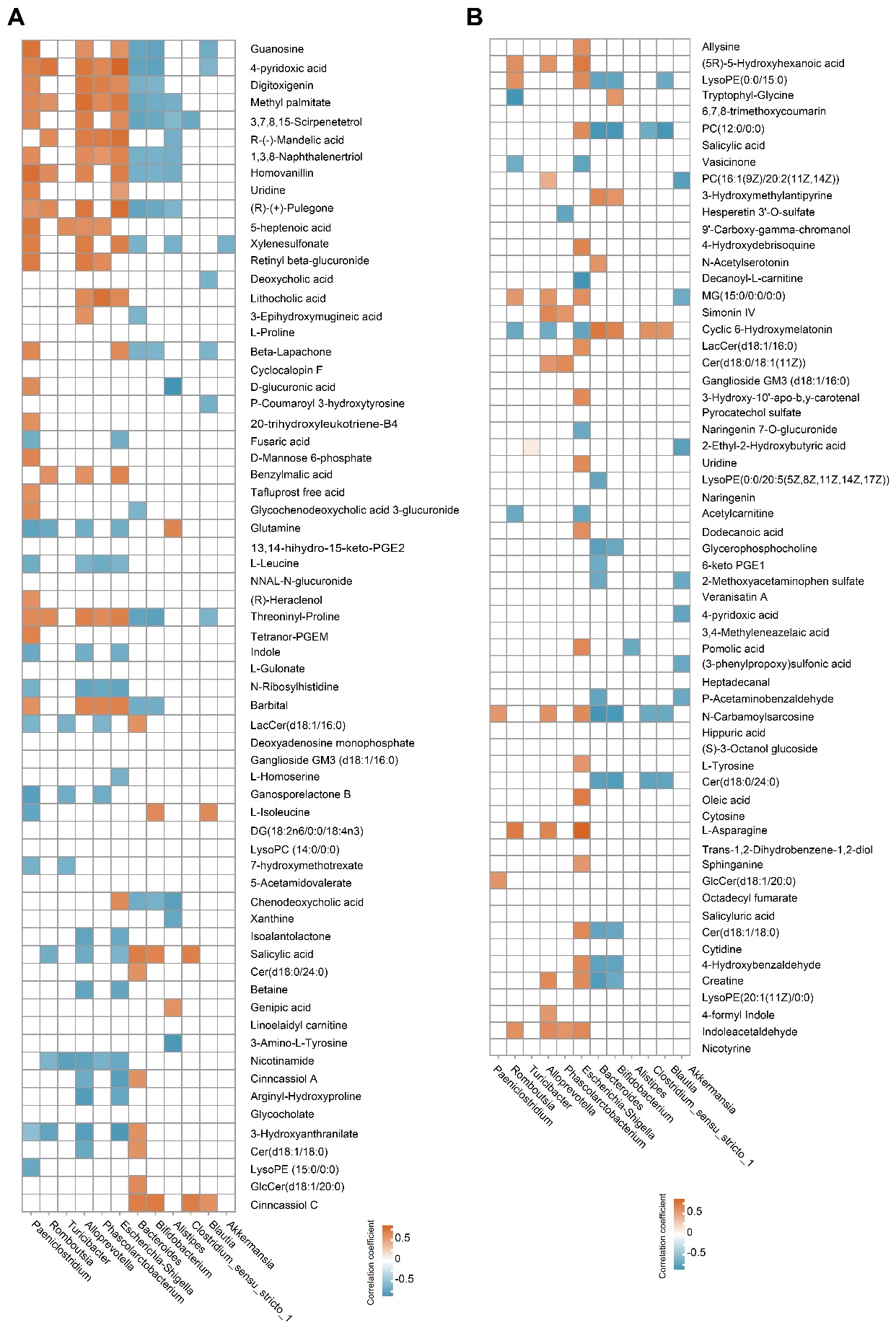


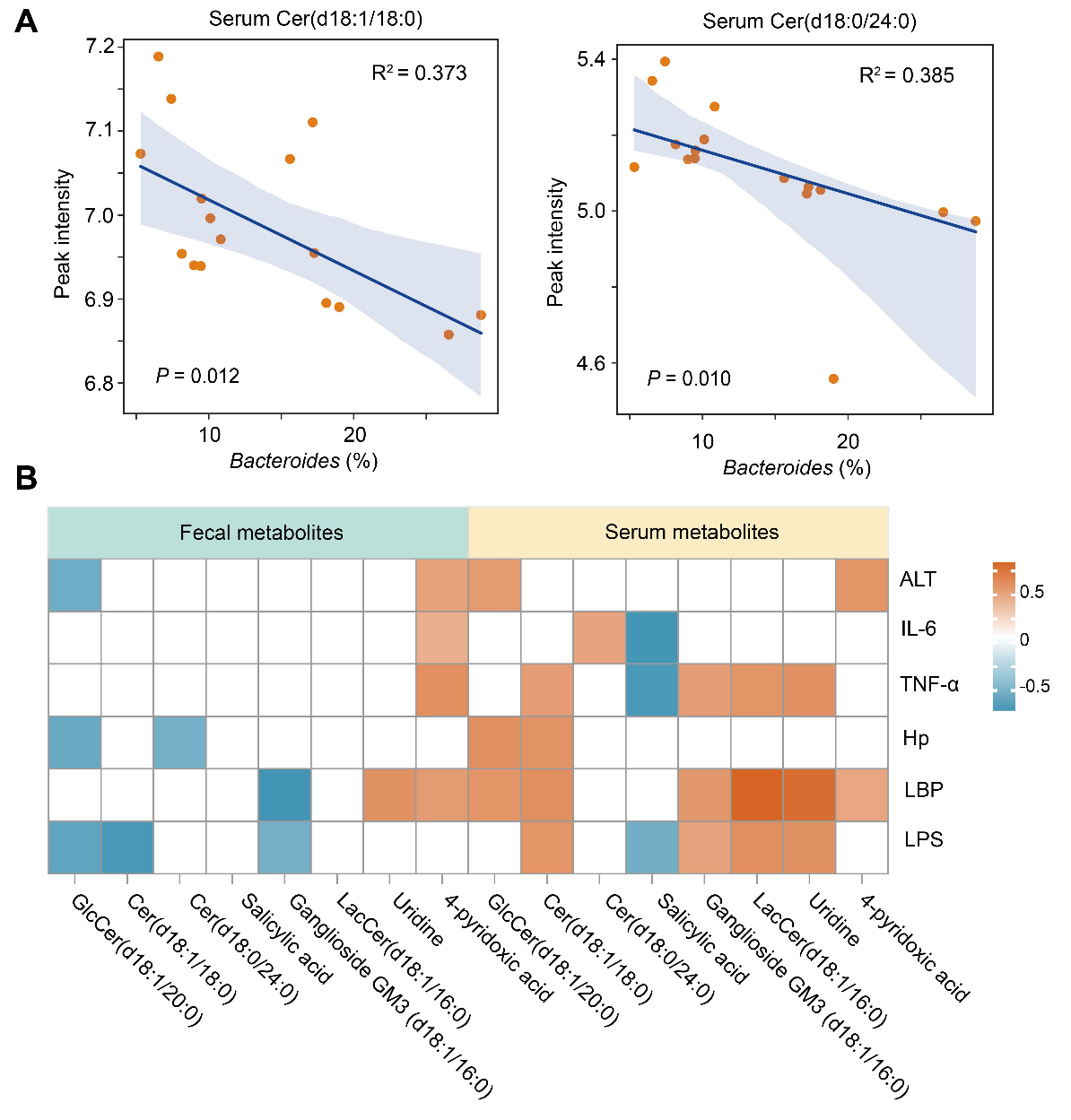


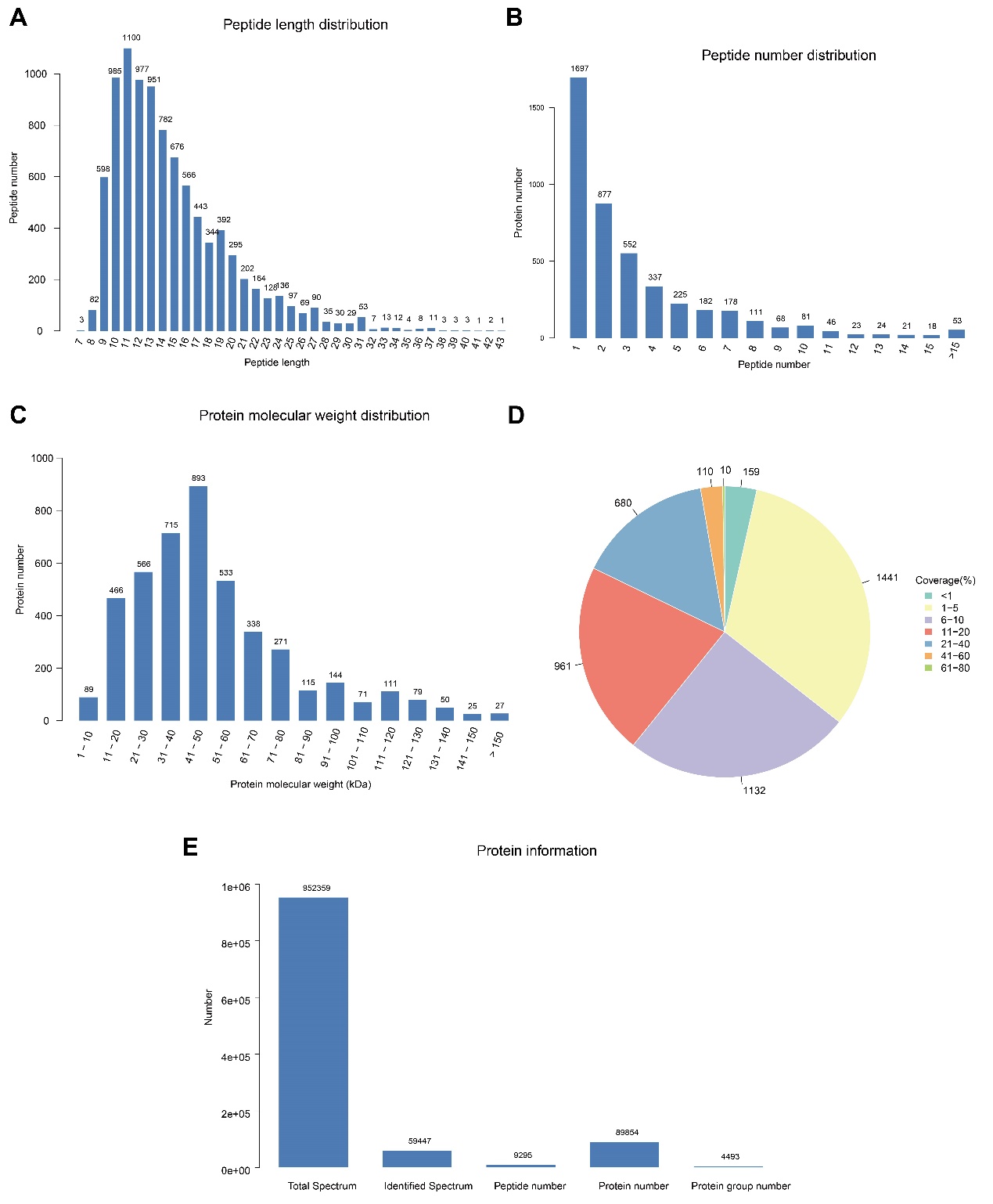


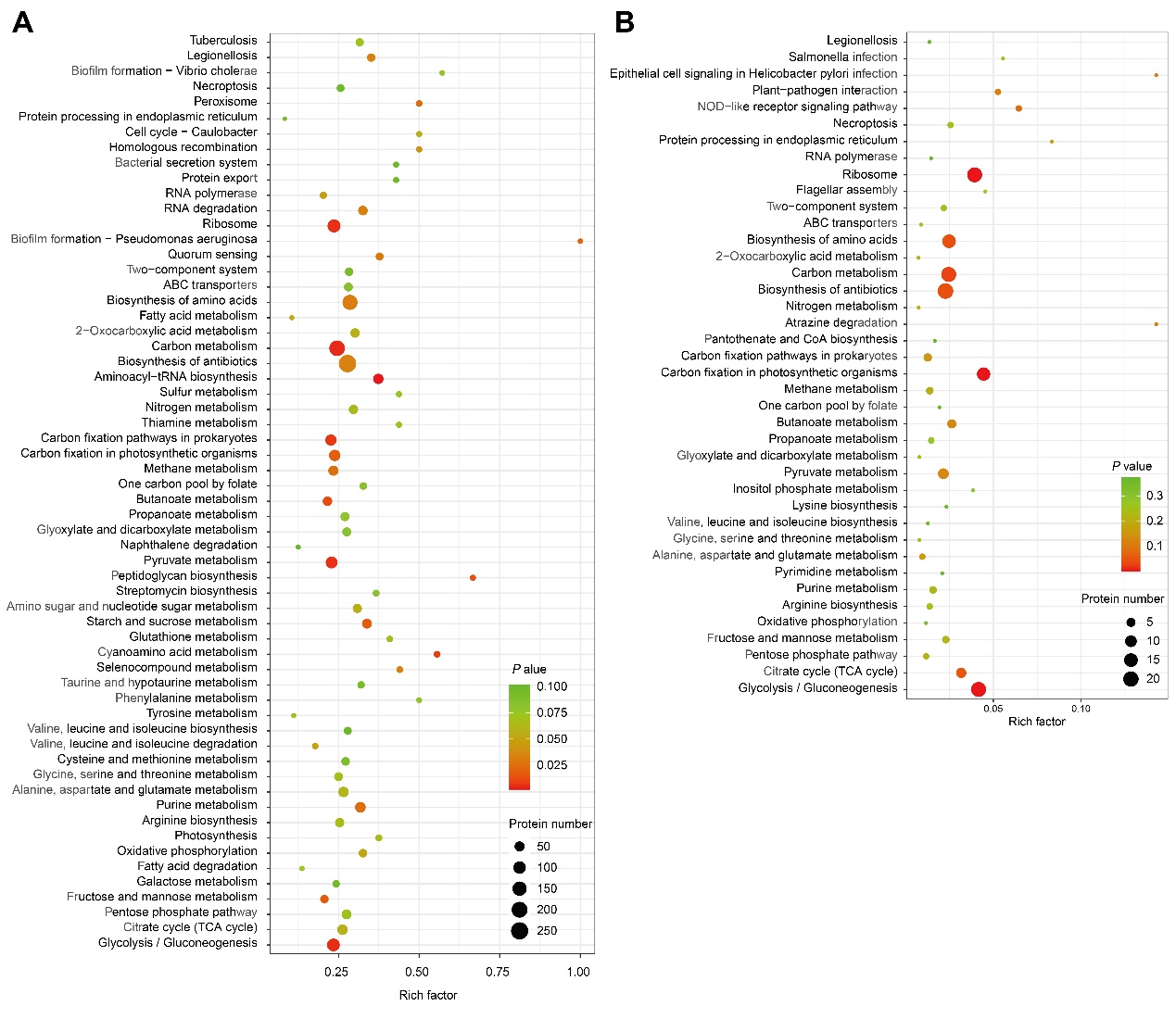

Supplement: Supplementary file 3 — Additional file 2: Fig. S1. Heatmap of association analysis between significantly altered fecal bacteria and differential metabolites in feces (A) and serum (B). Only features showing strong significant correlations (|r| > 0.5 and P < 0.05) were visualized. Fig. S2. Association analysis among fecal bacteria, differential metabolites, and serum biochemical parameters. (A) Regression lines showing the relationships between fecal Bacteroides and serum Cer(d18:1/18:0) and Cer(d18:0/24:0). (B) Heatmap of association between shared metabolites and serum biochemical parameters. Only features showing strong significant correlations (|r| > 0.5 and P < 0.05) were visualized. ALT, alanine aminotransferase; Hp, haptoglobin; LBP, LPS-binding protein; LPS, lipopolysaccharide. Fig. S3. Identification and quantitative evaluation of identified fecal proteins. (A) Distribution of proteins based on peptide length distribution. (B) Distribution of proteins based on the number of peptides. (C) Distribution of proteins based on molecular weight. (D) Distribution of identified protein sequences. (E) Protein information. Fig. S4. KEGG enrichment analysis of differentially expressed proteins (DEM). (A) Upregulated DEM. (B) Downregulated DEM. [file 40168_2023_1661_MOESM2_ESM.docx]
